# YTHDF1 mediates *N*‐methyl‐*N* ‐nitrosourea‐induced gastric carcinogenesis by controlling HSPH1 translation

**DOI:** 10.1111/cpr.13619

**Published:** 2024-03-05

**Authors:** Peng Song, Xiang Li, Shuai Chen, Yu Gong, Jie Zhao, Yuwen Jiao, Yi Dai, Haojun Yang, Jun Qian, Yuan Li, Jian He, Liming Tang

**Affiliations:** ^1^ Department of Gastrointestinal Surgery The Affiliated Changzhou No. 2 People's Hospital of Nanjing Medical University Changzhou China; ^2^ Nanjing Drum Tower Hospital, Affiliated Hospital of Medical School Nanjing University Nanjing China; ^3^ The Collaborative Innovation Center for Cancer Personalized Medicine, School of Public Health Nanjing Medical University Nanjing China; ^4^ The Key Laboratory of Modern Toxicology, Ministry of Education, School of Public Health Nanjing Medical University Nanjing China

## Abstract

YT521‐B homology (YTH) domain family (YTHDF) proteins serve as readers that directly recognise m6A modifications. In this study, we aim to probe the role of YTHDF1 in environmental carcinogen‐induced malignant transformation of gastric cells and gastric cancer (GC) carcinogenesis. We established a long‐term low‐dose MNU‐induced malignant transformation model in gastric epithelial cells. In vivo and in vitro experiments were conducted to validate the malignant phenotype and characterise the roles of YTHDF1 and its downstream genes in malignant transformation cells. Additionally, we explored downstream m6A modification targets of YTHDF1 using RNA‐sequencing, RNA immunoprecipitation, and proteomics analyses, and conducted validation experiments in cell experiments and clinical samples. Long‐term low‐dose exposure of MNU converted normal Gges‐1 cells into malignant cells. YTHDF1 mRNA and protein expression are increased in MNU‐induced malignant cells (*p*<0.001). Meanwhile, YTHDF1 knockdown inhibits the malignant potential of MNU‐treated cells (*p*<0.01). YTHDF1 knockdown specifically suppresses HSPH1 protein, but not RNA levels. RIP‐qPCR validates HSPH1 is the target of YTHDF1 (*p*<0.01). HSPH1 knockdown impairs the malignant potential of MNU‐induced transformed cells. The increased expression of the key regulatory factor YTHDF1 in MNU‐induced gastric carcinogenesis affects malignant transformation and tumorigenesis by regulating the translation of downstream HSPH1. These findings provide new potential targets for preventing and treating environmental chemical‐induced gastric carcinogenesis.

## Graphical abstract

We established an MNU‐induced malignant gastric epithelial cell in vitro model. YTHDF1 mRNA and protein expression are increased in MNU‐induced malignant gastric epithelial cells. Meanwhile, YTHDF1 knockdown inhibits the malignant potential of MNU‐treated cells. HSPH1 represents a downstream target of YTHDF1‐regulated translation; its knockdown impairs the malignant potential of MNU‐induced transformed cells.

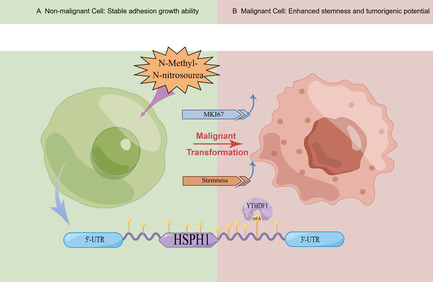

## 1 INTRODUCTION

*N*‐methyl‐*N*‐nitrosourea (MNU) is an alkylating agent that can exert carcinogenic activity without metabolic biological activation.^1^, ^2^ MNU is carcinogenic to the pancreatic, gastrointestinal, dental, reproductive, haematopoietic, immune, and sensory systems.^3^, ^4^, ^5^ It is a ubiquitous environmental carcinogen that can be acquired by humans through smoking, occupational exposure, and diet.^6^ MNU is a methylated nitrosourea compound that is typically formed from nitrite or creatinine in fermented food and methylurea in gastric juice.^7^ Moreover, given that MSU contributes to tumour invasion and metastasis by inducing DNA mutation and proto‐oncogene expression, it is commonly used to establish animal tumour models.^8^, ^9^ In addition, MNU induces abnormal angiogenesis and contributes to tumour development.^10^ For instance, in renal cell carcinoma, MNU induces the formation of DNA adduct O6 methylguanine in mesenchymal stromal cells and renal cortical tubular cells.^11^ Meanwhile, in gastric cancer (GC), epigenetic changes exert greater impacts on the risk of cancer development than genetic changes.^12^ In fact, numerous studies have revealed that nitroso compounds represent risk factors for GC. Various cell signalling pathways, including inflammatory pathways, cell proliferation, and DNA repair, are involved in the development of nitroso compound‐related gastrointestinal tumours.^13^, ^14^, ^15^

N6‐methyladenosine (m6A) represents the most prevalent modification in mammalian mRNA and non‐coding RNA, playing vital roles in gene transcription, protein interactions, as well as mRNA splicing, stability, translation, and metabolism.^16^, ^17^ The distribution of m6A sites is not random and is frequently present within 3′‐untranslated regions (UTRs).^18^, ^19^ Moreover, m6A methylation is regulated by appointed enzymes classified as methyltransferases (‘writers’, e.g., methyltransferase‐like 3(METTL3)), demethylases (‘erasers’, e.g., alpha‐ketoglutarate dependent dioxygenase (FTO) and alkylation repair homologue protein 5 (ALKBH5)), and m6A‐binding proteins (‘readers’, e.g., YTHDF1‐3 and YTHDC1, 2). Several studies have reported that upregulation of total m6A methylation^20^ and METTL3 abundance? representative m6A methyltransferase—promote? GC development.^21^, ^22^ In contrast, lower expressions of FTO and ALKBH1 are associated with significantly improved prognosis in patients with GC.^23^ Additionally, the ‘readers’, which directly recognise m6A modifications, include YT521‐B homology (YTH) domain family (YTHDF) proteins (YTHDF1‐3 and YTHDC1, 2). YTHDF2 recognises m6A sites on targeting mRNAs and regulates their decay.^17^ Additionally, through binding to translation initiation factors, YTHDF1 recruits the small 40S ribosome subunit to mRNA to mediate cap‐independent mRNA translation.^17^ Similarly, YTHDF3 facilitates mRNA translation or degradation in cooperation with the other two YTHDFs. In fact, YTHDF3 shares numerous targets with YTHDF1 (58%) and YTHDF2 (60%). Although inhibition of YTHDF1 expression reportedly interferes with GC progression,^24^, ^25^ the involvement of m6A methylation in MNU‐induced gastric carcinogenesis, as well as the associated modification factors and downstream target genes, remains largely unknown.

Assessing the carcinogenic potential of MNU is necessary for human health risk estimation. Carcinogenicity assays in experimental animals are considered the ‘gold standard’ for evaluating chemical carcinogenicity. However, animal experiments are expensive and do not permit the simultaneous screening of multiple compounds. This has prompted the development of cell conversion assays in vitro. The transition of premalignant cells to malignant cells is a multistep process requiring various cellular alterations, including (1) immortalization and unlimited self‐renewal, (2) changes in morphology (e.g., acquisition of a spindle morphology) (3) altered growth patterns, (4) anchorage‐independent growth (e.g., cells forming colonies in soft agar), and (5) the ability to undergo tumour formation in vivo (e.g., tumour formation in nude mice).^26^

Our research provides a new cell model in vitro for the study of the molecular mechanism of gastric carcinogenesis, and on this basis, explores the role of YTHDF1 in the process of gastric carcinogenesis.

## 2 MATERIALS AND METHODS

### 2.1 Transcription data analysis

The Cancer Genome Atlas‐Stomach Adenocarcinoma (TCGA‐STAD) cohort and three GC cohorts (GSE57303, GSE15459, and GSE62254/ACRG) from gene s (GEO) databases were included in the analysis (Table S1, baseline information for the GC datasets). The data were analysed using R (version 4.1.2) and R Bioconductor packages.

### 2.2 Cell culture

The human gastric mucosal epithelial cell line, GES‐1, was procured from the Chinese Academy of Sciences cell bank and cultured in Gibco's Dulbecco's modified eagle medium (DMEM; New York, NY, USA) containing 10% fetal bovine serum (FBS; Gibco, New York, USA), 100 μg/mL streptomycin, and 100 U/mL penicillin? streptomycin (Gibco) at 37°C and 5% CO_2_. For model establishment, GES‐1 cells were cultured in MNU (0.01 g/L) for 6 months. Non‐treated cells served as a control.

### 2.3 Cytotoxicity assay

The toxicity of MNU was measured using a Cell Counting Kit‐8 (CCK‐8; ck04, Dojindo, China). Adherent cells, grown in culture media enriched with 10% FBS, were seeded in 96‐well plates at 1 × 10^3^ cells/well and treated with various concentrations of MNU (10, 10^2^, 10^3^, 10^4^, 10^5^, 10^6^, 10^7^, 10^8^, and 10^9^ ng/L) for 24–72 h. On day 3, 10 μL of CCK‐8 (5 mg/mL) was added to each well, and the absorbance was measured at 450 nm using a microplate reader (Versamax microplate reader, Molecular Devices, CA, USA).

### 2.4 Cell proliferation, migration, and invasion assays

To assess cell proliferation, 2 × 10^4^ cells/well were seeded into 6‐well plates. On days 1, 2, and 3, the cells were counted with a haemocytometer according to the manufacturer's directions, and a standard curve was generated. The experimental treatments were performed in triplicate.

A Transwell chamber assay (Corning Costar Corporation, Corning, New York, NY, USA) was used to assess cell migration. Briefly, the Transwell upper chambers were soaked in media without serum overnight. The medium was then discarded, and 5 × 10^3^ cells were seeded in the upper chambers in DMEM medium and allowed to adhere. To the lower chambers, 750 μL of medium containing 20% FBS was added. After 48 h of culture, 4% cold formaldehyde containing 0.05% crystal violet (MCE China, Shanghai, China) was added to the bottom of the upper chamber to fix the cells.

Invasion assays were performed by applying 20% Matrigel (BD Biosciences, San Jose, CA, USA) to the surfaces of the upper Transwell chambers. All subsequent steps were the same as those for the migration assay.

### 2.5 Colony formation assay

A 6‐well plate was seeded with 1 × 10^3^ cells in 2 mL of culture medium per well. After 2 weeks of culture without changing medium, 4% cold formaldehyde containing 0.05% crystal violet was applied to the bottom to fix the colonies.

### 2.6 Reverse transcription‐quantitative (RT‐q) PCR

Total cellular RNA was extracted using TRIzol (TaKaRa, Nojihigashi, Japan) and quantified using the QuantiFluor RNA System (Promega, Madison, Wisconsin, USA) and reverse‐transcribed into first‐strand cDNAs with a reverse transcription kit (Beyotime Biotechnology, Jiangsu, China). The abundance of cDNA was measured with the use of Sybr Green using a HT‐7900 RT‐qPCR system (Applied Biosystems, Carlsbad, CA, USA). qPCRs were run using SYBR mix (Beyotime Biotechnology, Jiangsu, China) and a Thermal Cycler (C1000 Touch, Bio‐Rad, Hercules, CA, USA). The thermal cycling programme was as follows: 50°C, 2 min; 95°C, 10 min; 40 cycles of 15 s at 95°C; and 60°C, 1 min. All experiments were performed in triplicate and repeated three times. The relative expression levels were determined based on the threshold cycle (Ct) and correlated with the control group. For long‐term storage, amplificated samples were stored at −30°C to −15°C. *ACTB* (β‐Actin) was used as the endogenous control. Primer sequences were determined using established gene sequences (Table S2).

### 2.7 Western blot analysis

Cellular proteins were isolated using Radio Immunoprecipitation Assay (RIPA) buffer (Beyotime Biotechnology, Jiangsu, China) on ice and centrifuged to collect supernatants for western blotting (12,000 × *g*, 10 min). Protein quantification was performed using a bicinchoninic acid kit (Beyotime Biotechnology, Jiangsu, China). The quantified samples were separated by electrophoresis on 10% sodium dodecyl sulphate polyacrylamide gels and transferred to nitrocellulose membranes. Membranes were blocked with 3% milk in Tris buffer saline (TBS)‐0.5% tween (TBS‐T) for 1 h at room temperature, washed thrice with TBS‐T and incubated with primary antibodies diluted in TBS to 1:1000 at 4°C overnight. The following primary rabbit polyclonal antibodies were used: β‐actin (ab8226, Abcam, Cambridge, UK), YTHDF1 (ab252346, Abcam, Cambridge, UK), YTHDF2 (ab220163, Abcam, Cambridge, UK), YTHDF3 (ab220161, Abcam, Cambridge, UK), HSPH1(ab109624, Abcam, Cambridge, UK). Subsequently, the membranes were rinsed and incubated with horseradish peroxidase‐conjugated secondary antibodies (anti‐rabbit IgG,1:5000, Cat. No. 31460), anti‐mouse IgG (1:5000, Cat. No.31430; Thermo Fisher Scientific, Rockford, IL, USA) at room temperature for 1 h. Protein bands were quantified using Quantity One software version 4.6.6 (Bio‐Rad, Hercules, CA, USA).

### 2.8 Immunofluorescence

A 20‐mm diameter glass‐bottom cell culture dish (801,001; Nest Biotechnology, Shanghai, China) was seeded with 1 × 10^3^ cells in 2 mL of culture medium. After 48 h of culture, 4% paraformaldehyde was applied to the dish for 30 min at room temperature to fix the cells; cell membranes were permeabilized in 0.5% Triton X‐100 for 15 min. At room temperature, the cells were blocked in 1× phosphate‐buffered saline (PBS) with 1% goat serum in for 1 h to avoid nonspecific binding. The blocking solution was discarded, and cells were washed thrice with PBS and incubated with primary rabbit anti‐YTHDF1 (1:100, ab252346, Abcam, Cambridge, UK) at 4°C overnight. Following three rapid washes, the primary antibody were recycled and cells were incubated with goat anti‐rabbit IgG‐488 (A‐11034; Thermo Fisher Scientific, Rockford, USA) at 37°C for 1 h. Finally, the nuclei were stained with 5% DAPI (Cell Signalling Technology, Danvers, MA, USA) for 5 min. The cells were analysed under an inverted fluorescence microscope (IX7; Olympus, Tokyo, Japan).

### 2.9 Cell live/dead assay

To assess cell viability, cells were stained with live/dead stain using a Live/Dead Cell Double Staining Kit (Sigma‐Aldrich, St. Louis, MO, USA) according to the manufacturer's instructions. Cells were incubated with live/dead staining solution and incubated for 30 min. The proportion of living cells to dead cells was determined by measuring fluorescence at 490 nm and 540 nm? respectively, under a confocal microscope.

### 2.10 Soft agar assay

To measure anchorage‐independent growth in vitro, soft agar colony formation assays were performed. A 1:1 mixture of medium containing 20% FBS and 1.2% soft agar (prepared using PBS) was applied to 6‐well plates at 2 mL/well. The plates were incubated at 37°C for 30 min to solidify the agar. Cells were counted and added to a 1:1 mixture of 0.6% soft agar and double‐strength medium containing 20% FBS. The mixture was added to 6‐well plates at 1.5 mL/well. Every 48 h, 200 μL of culture medium was applied per well to supplement the volatilised media. Colonies with more than one cell were counted.

### 2.11 Suspension 3D culture

Suspended spheres were cultured in 96‐well round‐bottom ultra‐low attachment plates (Corning, New York, NY, USA) to prevent cell attachment. Cells were seeded at 2.0 × 10^3^ cells per well on day 1 in a final volume of 100 μL. The medium containing 10% FBS was changed every 4 days. The experiment ended on the 14th day of culture and sphere size was monitored regularly throughout the study period.

### 2.12 Subcutaneous xenograft tumour model establishment

We established subcutaneous xenograft tumour models with 6‐week‐old female BALB/c nu/nu mice (Cavens Laboratory Animal, Chang Zhou, China) to assess the tumorigenic property of MNU in vivo. All mice were acclimatised for 1 week before operation and subsequently stored in the following environment until sacrifice: constant temperature and humidity, 12 h light/dark cycle under specific pathogen‐free conditions with ad libitum access to food and water. Mouse experiments were conducted in compliance with international animal care and maintenance regulations and were approved by the Animal Ethics Committee of the Laboratory Animal Center of Nanjing Medical University (IACUC22‐0103). In brief, MNU‐induced or control GES‐1 cells were injected subcutaneously (3 × 10^6^ in 100 μL PBS/mouse) into the groins of mice which were separated into two equisized groups (*n* = 6 per group). The mice were continuously monitored and sacrificed by breaking the neck 1 week after tumours were first detected.

### 2.13 Retroviral infection, transfection, and construction of stable knockdown cell lines

The shRNA (GeneChem Co., Ltd, Shanghai, China) target sequences were: *YTHDF1*: 5′‐CCCGAAAGAGTTTGAGTGGAA‐3′; 5′‐GTTCGTTACATCAGAAGGATA‐3′; *HSPH1*: 5′‐CCAGTAACAGATTGTGTTATT‐3′; 5′‐ATGAGAAATACAACCATATTG‐3′. To construct stable *YTHDF1*/*HSPH1* knockdown cells, MNU‐induced malignant GES‐1 cells were cultured in the harvested lentivirus‐containing supernatant for 48 h and screened for successful lentiviral transfection in medium with 2 μg/mL puromycin for 3 days; media was replaced each day. Knockdown was confirmed by western blotting.

### 2.14 Proteomics analysis

Cellular proteins were isolated using RIPA lysis buffer on ice. Cell lysates were centrifuged for 10 min (12,000 × *g*) and the cellular pellet was discarded. As per a previously described procedure,^27^ proteins were digested with trypsin. The peptides were then desalted and reconstituted using an Empore™ SPE C18 cartridge (standard density, Sigma, Aldrich, Saint Louis, MO, USA), within 0.1% (v/v) formic acid.

LC–MS/MS analysis was carried out by using a timsTOF Pro mass spectrometer (Bruker Daltonics, Bremen, Germany). Protein quantification and identification were performed using the MaxQuant software (version 1.5.3.17). Hierarchical clustering analysis was performed with Java Treeview software (http://jtreeview.sourceforge.net) and Cluster analysis of phosphorylated peptides 3.0 (http://bonsai.hgc.jp/mdehoon/software/cluster/software.htm). Motifs analysis was performed with MeMe (http://meme-suite.org/index.htm). The CELLO (http://cello.life.nctu.edu.tw/) multiclass support vector machine classification system was used to predict protein subcellular locations. Protein sequences were searched by using InterProScan software to identify protein domains in the InterPro member database Pfam. Gene enrichment analysis was performed with Fisher's exact test which used the entire quantitative protein as the background data set. STRING (https://string-db.org/), was used for protein interaction analysis.^28^ Gene Ontology (GO, c5.go.v7.4.symbols.gmt) and Kyoto Encyclopedia of Genes and Genomes (KEGG, c5.go.v7.4.symbols.gmt) gene sets were downloaded from the MSigDB database (http://www.gsea-msigdb.org/gsea/index.jsp) to perform GSVA analysis.^29^

### 2.15 RNA‐sequencing (RNA‐seq)

Cellular RNA was extracted by TRIzol. Eukaryotic mRNA was enriched from the qualified samples using Oligo(dT) magnetic beads (Thermo Fisher Scientific, Rockford, IL, USA). Using the mRNA as a template, the first‐strand cDNA was synthesised with six‐base random primers. Next, the second‐strand cDNA was synthesised using buffer, dNTPs, and DNA polymerase I. The final cDNA library was obtained by PCR enrichment. CASAVA base call analysis was used to convert the original image data files, obtained through high‐throughput sequencing, to the original sequencing reads. FeatureCounts software was used to calculate the expected number of fragments per kilobase and per million base pairs of transcript sequence.

### 2.16 Paraffin embedding

Tissues were fixed in 4% paraformaldehyde and dehydrated using an ethanol gradient (JJ‐12 J, Wuhan Junjie Electronics, Wuhan, China) comprising 75%, 85%, 90%, 95% alcohol for 2, 2, 1.5, 2 h, respectively, and cleared with xylene (Sinopharm Chemical Reagent Co., Ltd. Shanghai, China) for 40 min. The tissues were embedded in paraffin (JB‐P5, Junjie Electro Co., Wuhan, China) and cooled in a − 20°C freezing table (JB‐L5, Junjie Electro Co., Wuhan, China). Tissues sections were then cut into 4 μm slices using a microtome (RM2016, Leica Instruments Co., Ltd., Shanghai, China). Finally, sections were flattened by floating them in 40°C warm water within the spreader (KD‐P, Zhejiang Jinhua Kedi Instrumental Equipment Co., Ltd. Zhejiang, China) and baked at 60°C. The flattened tissue sections was stored at room temperature.

### 2.17 Haematoxylin & eosin (H&E) staining

Tissues sections were treated with xylene (Sinopharm Chemical Agent Co., Ltd. Shanghai, China) I and II for 20 min each followed by absolute ethanol I and II for 5 min each, and washed with pure water. After dewaxing, the washed slices were placed in the haematoxylin dye solution for 3–5 min, washed with water, immersed in the differentiation solution, and washed again with running water. The stained sections were then dehydrated with 85% gradient alcohol for 5 min and 95% alcohol for 5 min. Subsequently, tissue samples were placed in anhydrous ethanol I, II, and III for 5 min each, dimethyl I and xylene II for 5 min each, and sealed with neutral gum. Image acquisition was performed using inverted microscope (Model, IX70; Olympus Corporation, Center Valley, PA, USA).

### 2.18 RNA immunoprecipitation (RIP) assay

To validate the interaction between YTHDF1 and mRNA transcripts, the Imprint RIP Kit (Sigma, Aldrich, USA) was used. Protein A agarose beads (Roche, Basel, Switzerland) were pre‐bound to antibodies; a total of 1 × 10^7^ MNU‐induced malignant cells were harvested with RIPA buffer and immunoprecipitated with rabbit anti‐YTHDF1 (1:200, ab252346, Abcam, Cambridge, UK) or 5 μg rabbit polyclonal IgG overnight. Protein and RNA isolations were performed using equal proportions of the sample volume, respectively.

### 2.19 Statistical analysis

All experiments were performed in triplicate and repeated three times. Student *t*‐tests (two‐tailed unpaired *t‐*test) were performed when comparing two groups of data, while one‐way analysis of variance and Newman–Keuls post hoc test were used to compared three groups. The Spearman Rank Correlation test was used to assess the expression of RNA modification genes. The Kaplan–Meier method was applied to generate the survival curve, and the log‐rank test was used to identify the prognostic significance of observed differences. All *p*‐values were bilateral, and *p* < 0.05 was defined as statistically significant. All data were analysed using GraphPad Prism 5.0 software (GraphPad software, Inc.) or SPSS 16.0 software (SPSS Inc.) and expressed as mean ± standard deviation (SD).

## 3 RESULTS

### 3.1 Construction of an MNU‐induced malignant transformation cell model

**FIGURE 1:**
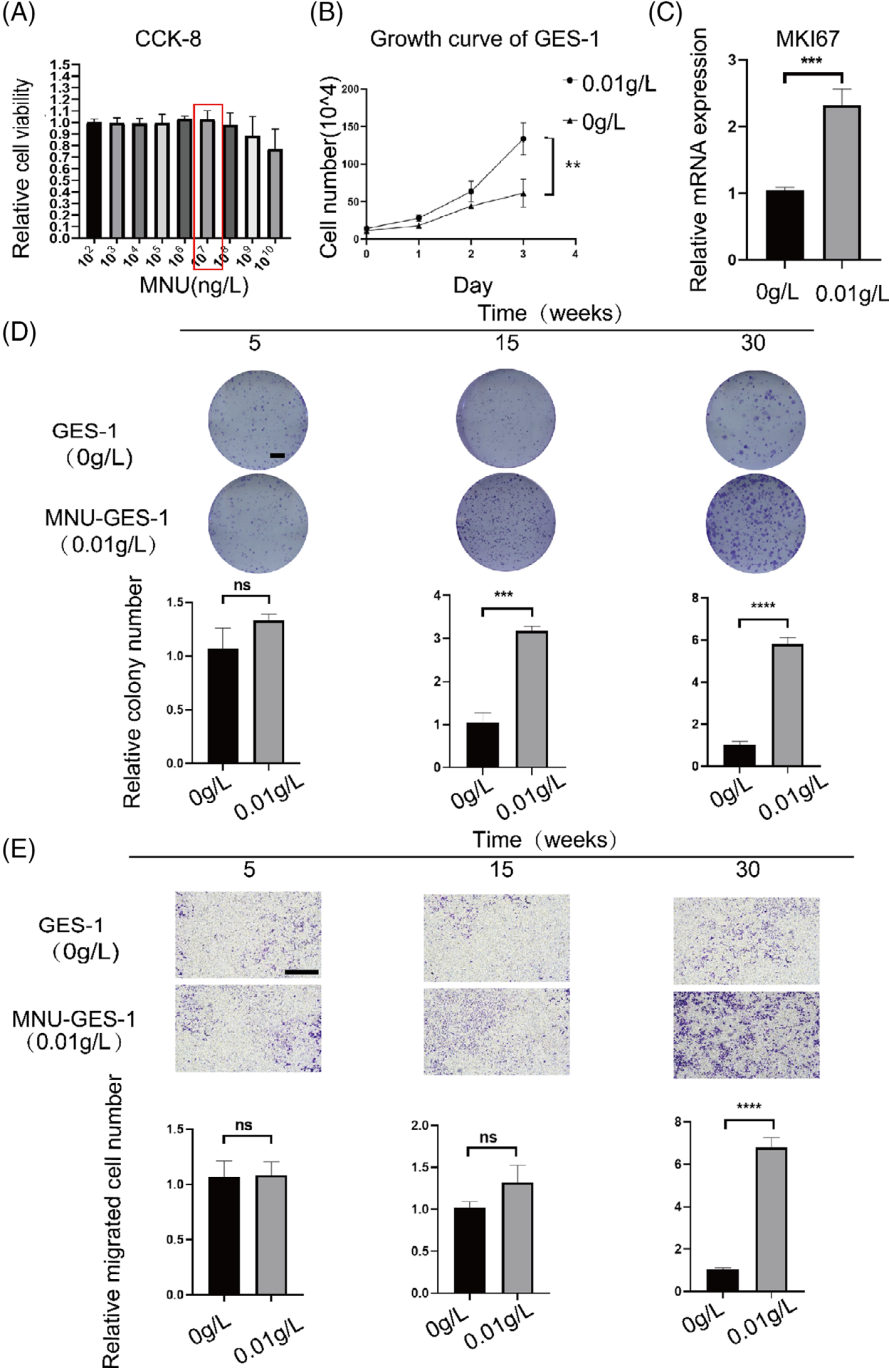
Long‐term MNU stimulation gradually induces changes in cellular biological processes. (A) Acute cytotoxicity of MNU measured using CCK‐8 assay. (B) Cell proliferation as monitored by cell counting in MNU‐induced and control GES‐1 cells. (C) *MKI67* mRNA expression in MNU‐induced and control GES‐1 cells. (D) Colony formation as determined by the colony formation assay and quantification of colony areas in MNU‐induced (1, 3, and 6 months) and control GES‐1 cells. E: Migration as assayed by the Transwell assay and quantification of migration areas of MNU‐induced (1, 3, and 6 months) and control GES‐1 cells. Images were taken at ×4 magnification. 0 g/L: control GES‐1 cells; 0.01 g/L: GES‐1 cells induced with 0.01 g/L MNU. ns *p >* 0.05, *p < 0.05, **p < 0.01, ***p < 0.001, ****p < 0.0001.

The results of the CCK‐8 kit cytotoxicity assay showed that the relative activity of GES‐1 cells treated with MNU was >95% at concentrations of 10^2^–10^7^ ng/L. Under these concentrations, short‐term stimulation of MNU had no effect on cell morphology (Figure 1A). Therefore, to mimic long‐term stimulation of the gastric mucosal epithelium by nitrosamines in the human body, we added 10^7^ ng/L (0.01 g/L) MNU to the medium to assess the long‐term low‐dose malignant transformation of GES‐1 cells in vitro (experimental group). GES‐1 cells cultured without MNU were used as a control. After stimulation for 6 months, the cell proliferation rate of the experimental group was significantly higher than the control group (Figure 1B) with the cell proliferation indicator MKI67 overexpressed in the experimental group (Figure 1C). Long‐term MNU stimulation also significantly enhanced the colony‐forming capacity of GES‐1 cells (Figure 1D). Meanwhile, the migration rate of cells gradually increased over time (Figure 1E) with no significant effect observed after 1 month of stimulation, and a significant increase was detected after 3 months.

**FIGURE 2:**
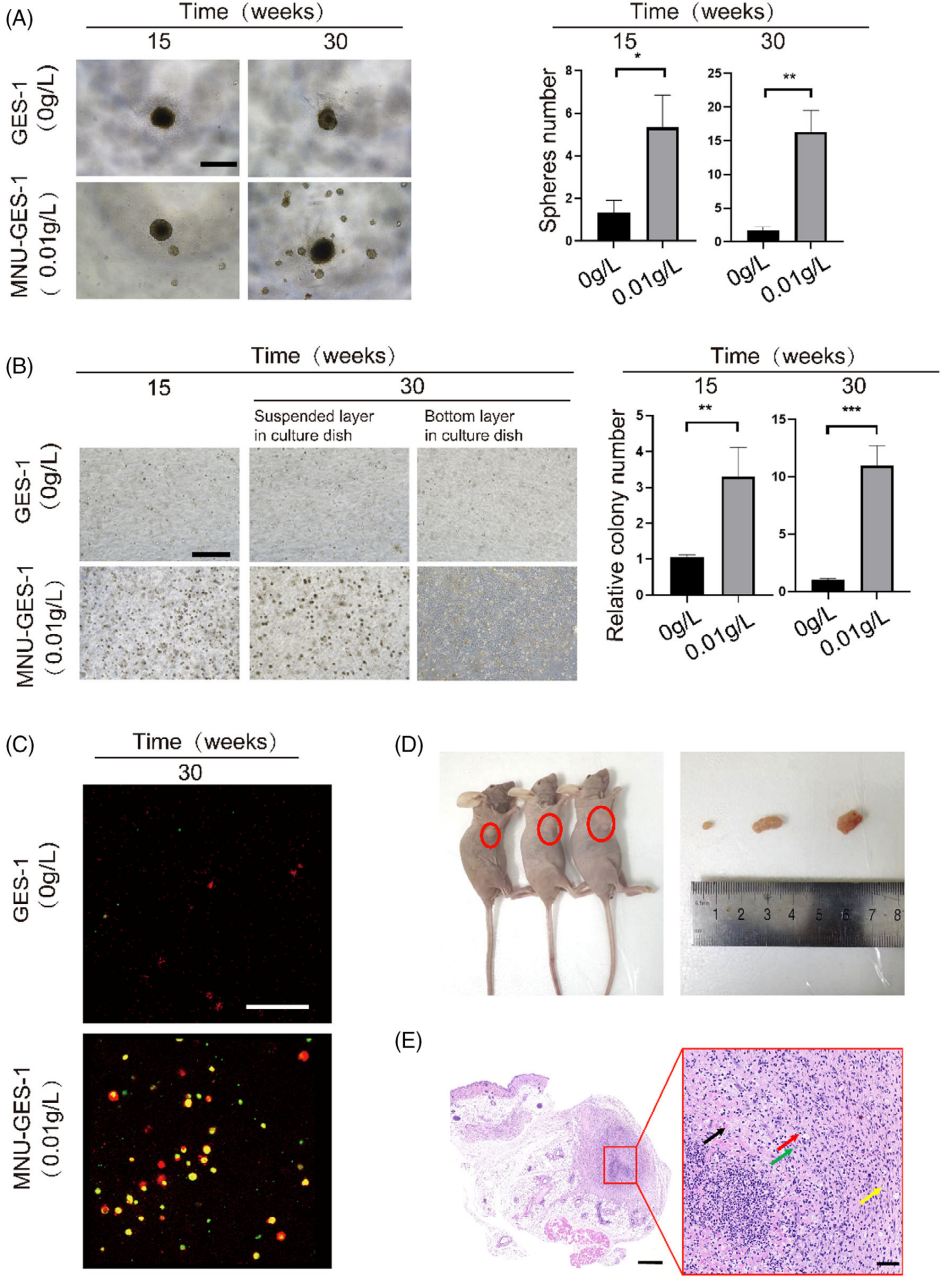
MNU‐induced gastric epithelial cell malignant transformation by long‐term low‐dose MNU induction. (A) Cell proliferation as assessed by a suspension spheroid formation assay in MNU‐induced (1, 4, and 6 months) and control GES‐1 cells. Images were taken at ×4 magnification. (B) Comparison of cell anchorage‐independent growth between MNU‐induced (1, 4, and 6 months) and control cells. Red box: cells growing adherent to the bottom of the well. Images were taken at ×4 magnification. (C) Live/dead cell staining showing viable (green) or dead (red) status of cells growth in soft agar. Images were taken at ×4 magnification. (D) MNU‐induced cell‐derived tumours (red circles) growing in nude mice and tumours were removed from the nude mice 4 weeks after inoculation. E: H&E staining of tumours. Black arrows: necrotic focus, massive cell necrosis, broken or dissolved nucleus, eosinophilic homogeneous cytoplasm; red arrows: infiltration of numerous lymphocytes and a few neutrophils; green arrow: surrounding connective tissue hyperplasia; yellow arrow: neovascularization. The lower right corner of the left image: black line = 500 μm; The lower right corner of the right image: black line = 50 μm. 0 g/L: control GES‐1 cells; 0.01 g/L: GES‐1 cells induced with 0.01 g/L. ns *p >* 0.05, *p < 0.05, **p < 0.01, ***p < 0.001, ****p < 0.0001.

Next, we performed 3D suspension cultures to assess the stem‐like properties and self‐renewal capacity of cells. The formation spheroids from single cells after several days of culture and the formation of new spheroids from single spheroid cells indicate stem cell characteristics.^30^ Through continuous analysis and confirmation of cells, we found that after 4 months of MNU stimulation, cells formed spheroids and the number and size of the spheroids increased significantly after culture up to 6 months (Figure 2A). These results demonstrated that these cells have self‐renewal potential. Anchorage‐independent growth is an important characteristic of cell transformation.^31^ Therefore, we examined the impact of MNU on GES‐1 cell proliferation in suspension culture. The proliferation rate of suspended cells increased significantly in soft agar (Figure 2B). A portion of the cells in the experimental group traversed two layers of soft agar and began to adhere to the bottom of dish. This phenomenon suggested invasion capacity, which is an important feature of transformed cells. In contrast, the proliferative and self‐renewal properties of control GES‐1 cells in suspension were poor (Figure 2B). To assess the status of the cells inside the soft agar colonies, we conducted live/dead cell staining. After culturing for 25 days, control cells stopped growing, whereas experimental cells continued to proliferate. The colonies in the experimental group were primarily composed of live cell clumps, whereas control cells gradually died after forming minor colonies (Figure 2C).

### 3.2 Effects of MNU on tumorigenicity in vivo

The tumorigenic ability of MNU‐induced cells in vivo was assessed in nude mice. GES‐1 cells are known to not be tumorigenic in nude mice. Four weeks after injection, subcutaneous tumour tissues were observed in mice administered MNU‐induced cells (Figure 2D). H&E staining revealed large necrotic foci and necrotic cells accompanied by infiltration of lymphocytes and neutrophils, along with numerous new blood vessels in the MNU‐induced cell‐derived tumours (Figure 2E). In nude mice inoculated with control GES‐1 cells, no primary tumours were observed. These data demonstrated that the transformed GES‐1 cell model induced by MNU was successfully established and that the cells had malignant potential.

### 3.3 Correlation between YTHDF1 protein and MNU‐induced malignant transformation cell model

To identify the critical regulatory factor in m6A methylation during MNU‐induced malignant cell transformation, we first conducted a bioinformatics analysis to detect potential GC risk factors. We selected 23 RNA‐modifying enzymes. In the 433 samples provided by TCGA, 22 m6A modification regulators were significantly upregulated in GC (Figure S1B). Univariate Cox regression analyses of TCGA‐STAD and GEO indicated that 6 of the 23 RNA modifiers were relevant to poor prognosis in GC (Figure S2). Next, to examine whether these six genes are related to MNU‐induced cell transformation, we compared their expression levels in the experimental and control groups. The mRNA level of *YTHDF1* was significantly higher after MNU stimulation (Figure S1A). Furthermore, acute drug exposure tests were conducted to explore the causality of the observed results. Following stimulation with different concentrations of MNU, the mRNA and protein levels of YTHDF1 increased in a dose‐dependent manner (Figure S1C).

**FIGURE 3:**
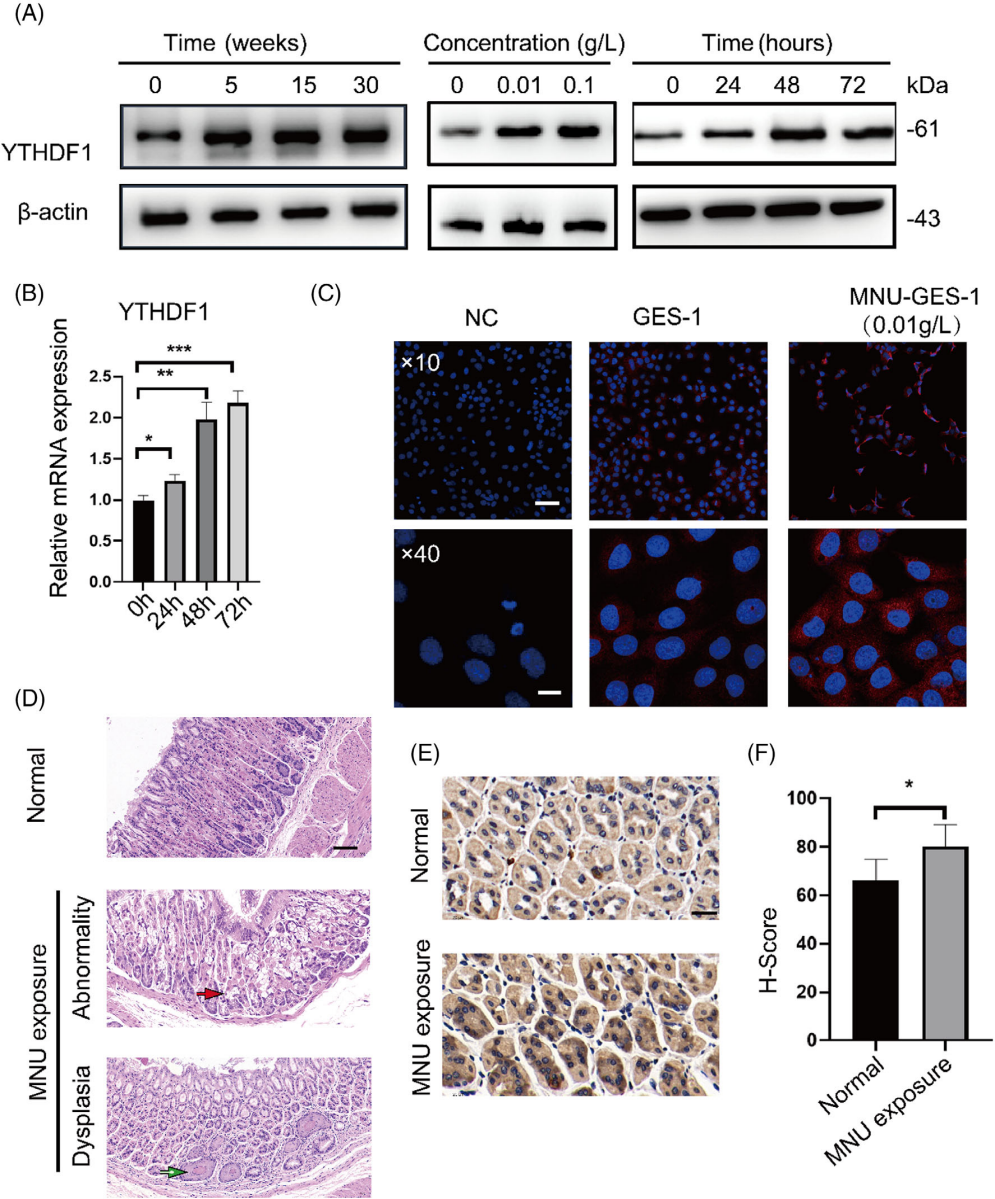
MNU‐induced YTHDF1 protein expressions were both time‐ and dose‐dependent. (A) Western blot analysis of YTHDF1 protein expression levels in GES‐1 cells induced by long‐term MNU at different times (0, 5, 15, 30 weeks), short‐term MNU at different doses (0, 0.01, 0.1 g/L), and short‐term MNU at different times (0, 24, 48, 72 h). (B) *YTHDF1* mRNA expression in GES‐1 cell lines induced by short‐term MNU at different times (0, 24, 48, 72 h). (C) Immunofluorescence assay of the YTHDF1 protein level of MNU‐induced malignant transformation cells and control GES‐1 cells. Blue staining represents nuclear DAPI staining, and red staining represents YTHDF1 protein staining. The above images were taken at ×10 magnification, white line = 250 μm. The above images were taken at ×40 magnification, white line = 50 μm. (D) Construction of MNU exposure mouse models. The above image shows H&E staining of the gastric tissue of the control group mice, while the middle and bottom images show H&E staining of the exposure group mice. The red arrow represents irregularly shaped gastric glands and mucous cells. The green arrow indicates gastric gland dysplasia. Black line = 100 μm. (E) Immunohistochemical staining on the gastric tissue of mice in the control and exposure groups to detect the level of YTHDF1 protein. Black line = 20 μm. (F) Comparative analysis of histochemical scores after immunohistochemical staining between the control group and exposure group mice. ns *p >* 0.05, *p < 0.05, **p < 0.01, ***p < 0.001, ****p < 0.0001.

Western blot results showed that YTHDF1 protein level increased in a dose‐dependent manner (Figure 3A), which was consistent with the transcriptional level results. Similarly, the levels of YTHDF1 RNA and protein increased in time‐dependent manner when GES‐1 was stimulated with 0.01 g/L MNU (Figure 3A, B). The YTHDF1 protein level was also consistent with the MNU induction time in long‐term MNU‐induced cells preserved for different periods of time. Immunofluorescence test showed that the abundance of YTHDF1 protein in the cytoplasm of malignant transformation cells increased (Figure 3C). Therefore, the expression level of YTHDF1 in malignant transformation cells is closely related to MNU induction.

To further investigate whether MNU exposure in vivo changes the expression of YTHDF1, we constructed MNU exposure mouse models (Figure 3D). Among the 10 cases in the exposure group, three exhibited atypical hyperplasia. The immunohistochemical results of mouse gastric tissue showed that the level of YTHDF1 in the exposure group was higher than that in the control group (Figure 3E, F). The above results indicate that MNU exposure in vivo can stimulate an increase in YTHDF1 expression, which is consistent with the trend of in vitro cell models.

### 3.4 YTHDF1 knockdown inhibits tumorigenicity

**FIGURE 4:**
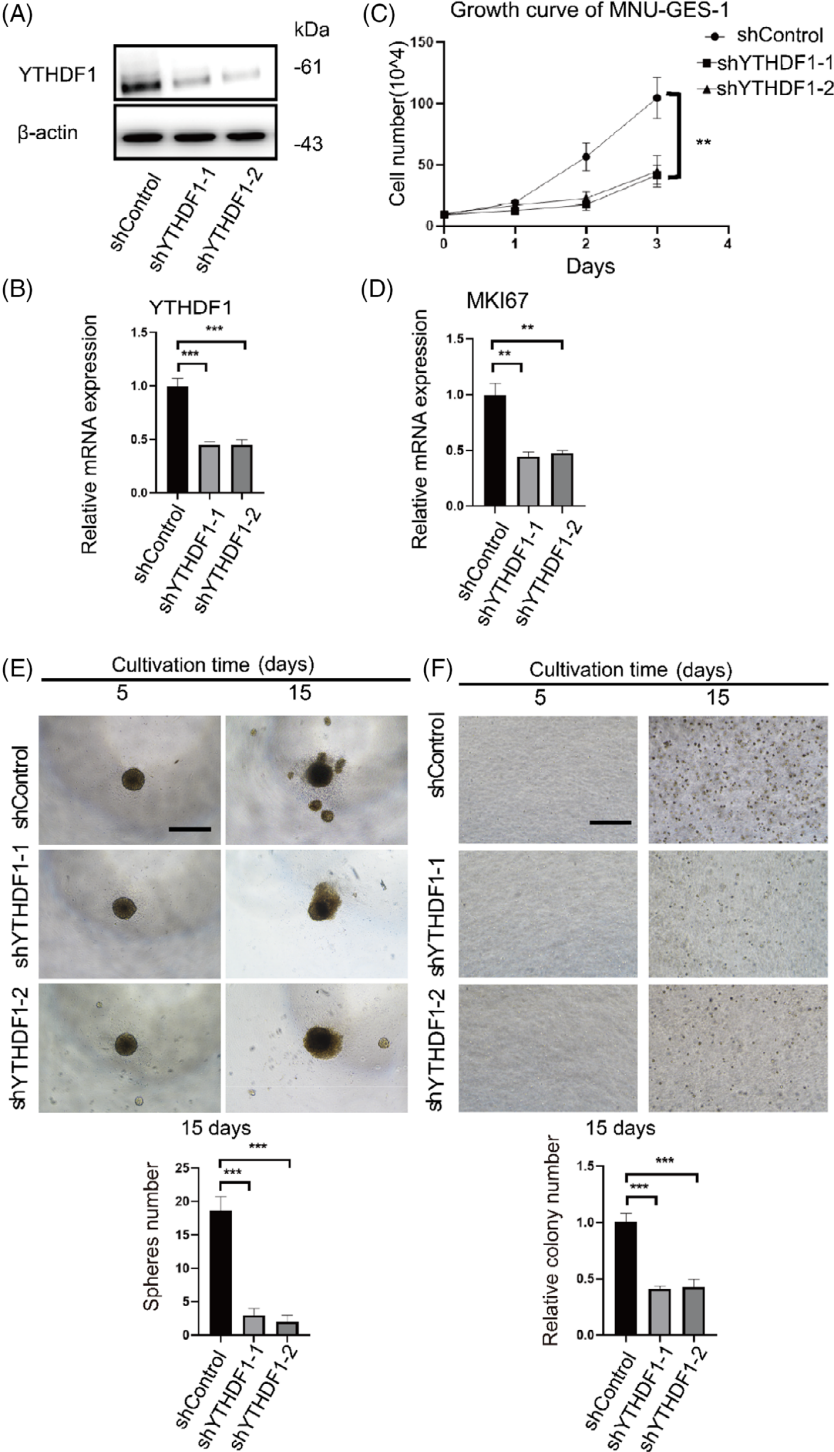
Effect of *YTHDF1* knockout on cell biological processes. (A) Western blot analysis of YTHDF1 abundance in MNU‐induced transformed cells infected with two independent shRNAs targeting *YTHDF1* (shYTHDF1‐1/2) or control shRNA (shControl). (B) *YTHDF1* mRNA expression in shControl transformed cells and shYTHDF1 transformed cells. (C) Cell proliferation of shControl transformed cells and shYTHDF1 transformed cells monitored by cell counting. (D) *MKI67* mRNA expression in shControl transformed cells and shYTHDF1 transformed cells. (E) Cell proliferation of shControl transformed cells and shYTHDF1 transformed cells in a suspension spheroid formation assay. The histogram compares the number of spheres between shControl and shYTHDF1 transformed cells. Black line = 500 μm. (F) Comparison of cell anchorage‐independent growth between shControl transformed cells and shYTHDF1 transformed cells. The histogram compares the number of colonies between shControl and shYTHDF1 transformed cells. Black line = 500 μm. ns = *p >* 0.05, *p < 0.05, **p < 0.01, ***p < 0.001, ****p < 0.0001.

To further explore whether YTHDF1 has a critical role in the malignant transformation cell model, we knocked down *YTHDF1* in the cell model using shRNA and confirmed successful knockdown by western blot and qPCR (Figure 4A, B). *YTHDF1* knockdown impaired the proliferation of transformed cells (Figure 4C). MKI67 expression increased during MNU‐induced cell transformation, however, decreased after gene knockdown (Figure 4D). Additionally, *YTHDF1* knockdown decreased spheroid numbers (Figure 4E) and suppressed the proliferation of transformed cells in suspended condition in soft agar colony formation assays (Figure 4F). These results indicated that *YTHDF1* knockdown negatively affected the stem cell potential and tumorigenicity of the MNU‐induced malignant transformed cells.

**FIGURE 5:**
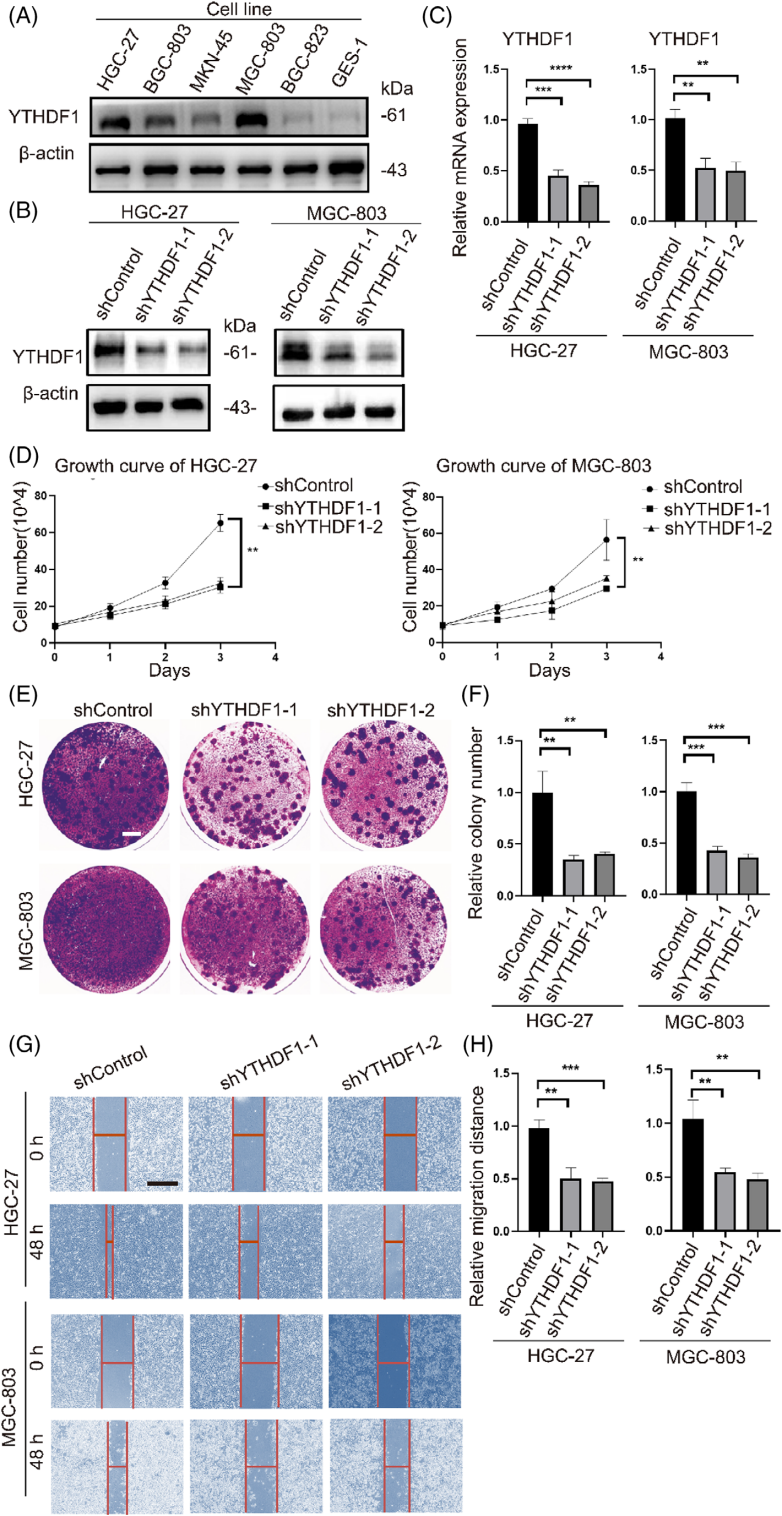
YTHDF1 knockdown in gastric cancer cells inhibits oncogenic ability. (A) Western blot analysis of YTHDF1 abundance in gastric cancer cells (HGC‐27, BGC‐803, MKN‐45, MGC‐803, and BGC‐823) and GES‐1. (B), (C) RT‐qPCR and Western blot analyses of YTHDF1 levels in HGC‐27/MGC‐803 cells infected with two independent shRNAs targeting *YTHDF1* (shYTHDF1‐1/2) or control shRNA (shControl). (D) The ability of cell proliferation in shYTHDF1‐1/2 and shControl groups of HGC‐27 and MGC‐803 cell lines resulted from cell counting assay. (E), (F) The clonogenic capacity in shYTHDF1‐1/2 and shControl groups of HGC‐27 and MGC‐803 cell lines resulted from colony formation assay. White line = 5 mm. (G), (H) The ability of cell migration in shYTHDF1‐1/2 and shControl groups of HGC‐27 and MGC‐803 cell lines resulted from scratch wound assay. Black line = 100 μm. ns = *p >* 0.05, *p < 0.05, **p < 0.01, ***p < 0.001, ****p < 0.0001.

The above results revealed that the knockdown of YTHDF1 suppressed the malignant phenotype of transformation cells. Next, we investigated the effect of YTHDF1 on the oncogenic capacity of GC cells. Through evaluating YTHDF1 levels in varieties of GC cells (HGC‐27, BGC‐803, MKN‐45, MGC‐803, and BGC‐823) and normal gastric epithelial cell GES‐1, we found YTHDF1 levels in GC cells increased compared with that in GES‐1 (Figure 5A). Among them, HGC‐27 and MGC‐803 cells had the highest YTHDF1 protein expression levels. We transduced shRNAs targeting YTHDF1 into HGC‐27 and MGC‐803 cells to knock down the expression of YTHDF1. The knockdown efficiency of YTHDF1 was verified by RT‐qPCR and Western blot (Figure 5B, C). Our in vitro findings indicated that YTHDF1 knockdown affected abilities of cell proliferation, migration, and clone formation (Figure 5D–H).

### 3.5 YTHDF1 regulates downstream target translation

**FIGURE 6:**
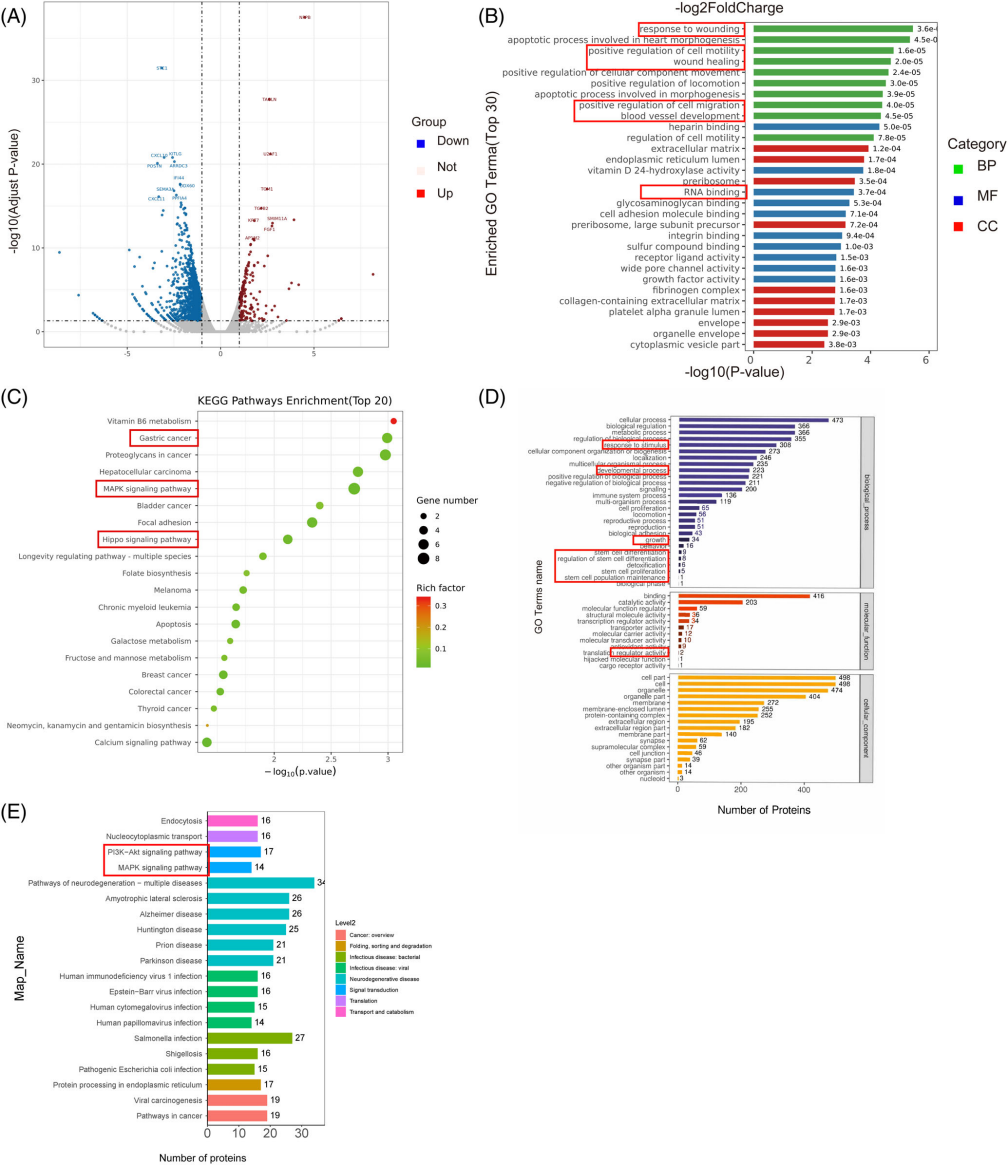
YTHDF1 is key to MNU‐induced transformation of GES‐1 cells. (A) Volcano plot of RNA‐seq. The lateral coordinate indicates the logarithmic value of the fold change in expression. The ordinate indicates the statistical significance of expression differences, expressed by *p*‐values. Each dot represents a specific gene; red and blue dots represent significantly up‐ and downregulated genes, respectively, and black dots represent genes that show no significant change. (B) GO term enrichment analysis. CC: cellular component, MF: molecular function, BP: biological process. (C) KEGG enrichment analysis. The 20 most enriched KEGG pathways are shown. Rich factor means proportion of differentially expressed genes in all genes. (D) and (E) GO and KEGG enrichment analyses of the genes upregulated among MNU‐induced malignant cells.

To further investigate MNU‐induced transformed cell properties and the complex role of YTHDF1, RNA‐seq analysis was conducted. Results revealed that various mRNA transcripts were dysregulated after MNU treatment in the experimental group (MNU‐induced vs. control GES‐1 cells, Figure 6A). GO term enrichment analysis indicated that upregulated genes include various biological processes, such as positive regulation of cell migration/movement, response to damage, and formation of blood vessels (angiogenesis) (Figure 6B). KEGG analysis revealed significant gene enrichment in the GC, MAPK, and Hippo signalling pathways (Figure 6C).

Next, we explored targets in YTHDF1‐involved cell transformation. As an m6A reader, YTHDF1 is not a regulator of gene transcription.^16^ We performed quantitative proteomics analysis of malignant transformed cells, control GES‐1 cells, and *YTHDF1*‐knockdown transformed cells. GO term enrichment analysis indicated that upregulated proteins in the experimental group were enriched in various biological pathways, including response to stimulation, proliferation, developmental process, stem cell regulation, and stem cell proliferation (Figure 6D). KEGG pathway enrichment analysis indicated that upregulated proteins in the experimental group were enriched in well‐known cancer‐related pathways, such as the MAPK and PI3k/Akt signalling pathways (Figure 6E). These results preliminarily revealed the mechanism of MNU in inducing cell malignant transformation and the role of YTHDF1 therein.

### 3.6 HSPH1 is a m6A‐modification target of YTHDF1 in MNU‐induced malignant transformed cells

To investigate the direct interaction between YTHDF1 and target transcripts, combined RNA‐seq and proteomics analysis was performed. We screened 179 genes with decreased protein expression in malignant transformed cells after *YTHDF1* knockdown, and 60,628 genes whose transcription levels did not change after *YTHDF1* knockdown. These two sets of genes were intersected and 51 candidate genes were considered for further analyses (Figure S3A). Using the TCGA dataset, the expression of these candidate genes in GC patients was analysed. The results indicated that 40/51 genes were significantly upregulated in tumours compared with adjacent normal tissues (Figure S3B). Univariate Cox regression analysis indicated that 10 genes (*BAG5*, *CSE1L*, *HSPH1*, *IPO7*, *MACF1*, *MYH9*, *MYH10*, *PMSD2*, *ROCK2*, and *RPS16*) were associated with poor tumour prognosis (Figure S3C). The DRACH motif, where D = G, A, or U; R = G or A; and H = C, A, or U, has been reported as a classic m6A motif.^32^ RNA m6A enrichment tends to be located at the 3′‐UTR and near‐stop codons.^19^ Using the SRAMP^33^ programme, we found that m6A peaks were located in the 3′‐UTRs of five candidate genes: *HSPH1*, *IPO7*, *USO1*, *MYH10*, and *ROCK2* (Figure S4A). A RIP qPCR assay indicated that, among these genes, *HSPH1* RNA was the most enriched in YTHDF1‐bound RNAs in malignant transformed cells (Figure S4B). To further verify whether YTHDF1 affects the expression of HSPH1 in clinical samples, we selected the pathological sections of 48 patients with GC undergoing surgery. Through immunohistochemical staining, we found that the abundance of HSPH1 protein was significantly correlated with that of YTHDF1 (Figure S5).

### 3.7 Inhibition of HSPH1 reduces tumorigenicity

**FIGURE 7:**
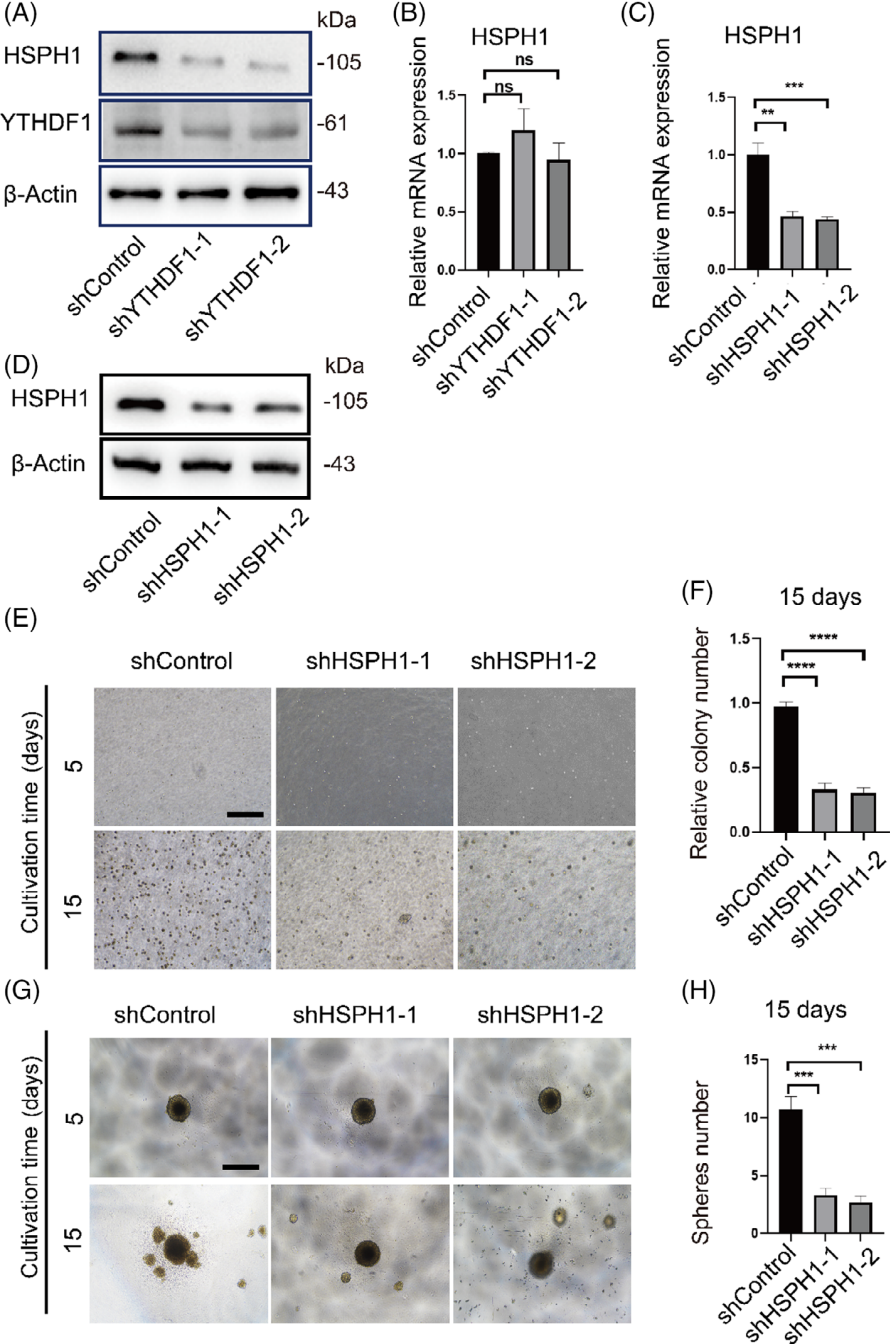
Inhibition of HSPH1 impairs the tumorigenic potential of transformed cells. (A) Western blot analysis of HSPH1 expression in transformed cells infected with two independent shRNAs targeting *YTHDF1* (shYTHDF1‐1/2) or control shRNA (shControl). (B) RT‐qPCR analysis of HSPH1 expression in transformed cells infected with two independent shRNAs targeting *YTHDF1* (shYTHDF1‐1/2) or control shRNA (shControl). (C), (D) RT‐qPCR and Western blot analyses of HSPH1 levels in transformed cells infected with two independent shRNAs targeting HSPH1 (shHSPH1‐1/2) or control shRNA (shControl). (E), (F) Comparison of cell anchorage‐independent growth between shControl transformed cells and shHSPH1 transformed cells. The histogram compares the number of colonies between shControl and shHSPH1 transformed cells. Black line = 500 μm. (G), (H) Cell proliferation of shControl transformed cells and shHSPH1 transformed cells in a suspension spheroid formation assay. The histogram compares the number of spheres between shControl and shHSPH1 transformed cells. Black line = 500 μm. ns = *p >* 0.05, *p < 0.05, **p < 0.01, ***p < 0.001, ****p < 0.0001.

To determine whether YTHDF1 affects HSPH1 expression by regulating HSPH1 translation, we performed RT‐qPCR and western blot analysis to measure the transcript and translation levels of HSPH1 after *YTHDF1* knockdown. As expected, *YTHDF1* knockdown reduced HSPH1 protein abundance, whereas the HSPH1 transcript level was not significantly affected (Figure 7A, B). Next, we explored whether HSPH1 affects the malignant potential of the cell model. We constructed stable *HSPH1* knockdown cell lines using lentivirus (Figure 7C, D). *HSPH1* knockdown suppressed the proliferation of transformed cells in suspension as well as the number of cell colonies (Figure 7E, F) and spheroids (Figure 7G, H).

**FIGURE 8:**
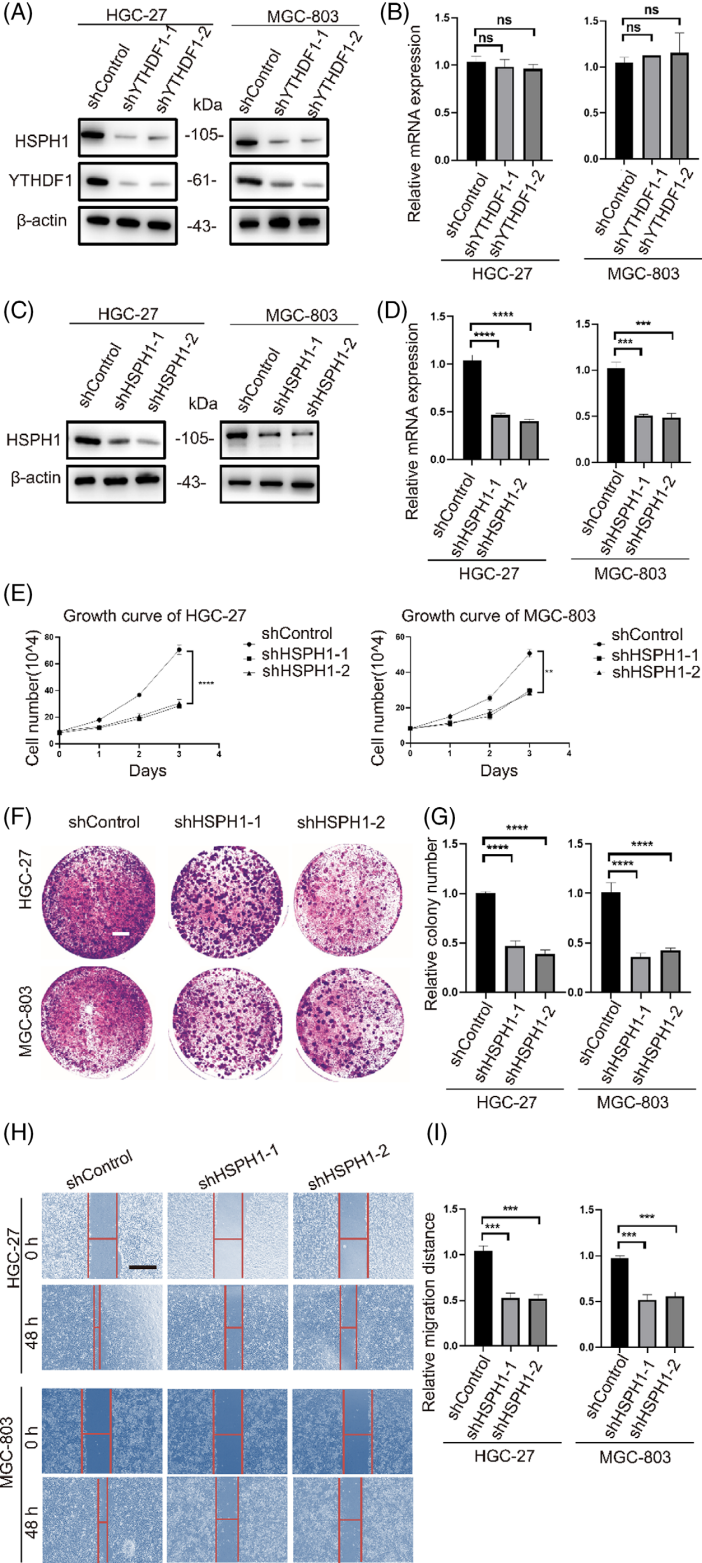
HSPH1 knockdown in gastric cancer cells inhibits oncogenic ability. (A), (B) Western blot and RT‐qPCR analyses of HSPH1 levels in HGC‐27/MGC‐803 cells infected with two independent shRNAs targeting *YTHDF1* (shYTHDF1‐1/2) or control shRNA (shControl). (C), (D) RT‐qPCR and Western blot analyses of HSPH1 levels in HGC‐27/MGC‐803 cells infected with two independent shRNAs targeting *HSPH1* (shHSPH1‐1/2) or control shRNA (shControl). (E) The ability of cell proliferation in shHSPH1‐1/2 and shControl groups of HGC‐27 and MGC‐803 cell lines resulted from cell counting assay. (F), (G)The clonogenic capacity in shHSPH1‐1/2 and shControl groups of HGC‐27 and MGC‐803 cell lines resulted from colony formation assay. White line = 5 mm. (H), (I) The ability of cell migration in shHSPH1‐1/2 and shControl groups of HGC‐27 and MGC‐803 cell lines resulted from scratch wound assay. Black line = 100 μm. ns = *p >* 0.05, *p < 0.05, **p < 0.01, ***p < 0.001, ****p < 0.0001.

The above results revealed that the repression of HSPH1 suppressed the malignant phenotype of malignant transformation cells. Next, we sought to identify whether YTHDF1 regulates HSPH1 protein expression in GC cells. RT‐qPCR and western blot analysis show that YTHDF1 regulates HSPH1 expression in GC at the translational level without affecting its mRNA expression (Figure 8A, B). We constructed stable HSPH1‐knockdown HGC‐27/MGC‐803 cell lines (Figure 8C, D). In vitro, results indicated that HSPH1 knockdown in HGC‐27 and MGC‐803 cells inhibited cell proliferation, migration, and clone formation in GC cells (Figure 8E–I).

## 4 DISCUSSION

Assessing the carcinogenic potential of chemicals is an important step in determining their risk to human health. The ‘gold standard’ method for assessing carcinogenicity is to perform carcinogenicity tests in experimental animals. However, given that in vivo experiments are time‐consuming and expensive, pose ethical issues, and do not allow high‐throughput screening of compounds, various in vitro cell transformation methods have been developed.

High intake of nitroso compounds may increase the risk of GC.^34^, ^35^ Compared with the damage caused by short‐term high‐dose exposure, that caused by long‐term low‐dose exposure is more representative of the actual situation and better mimics the process of nitroso compounds inducing human GC. Therefore, we induced gastric mucosal epithelial cell transformation using long‐term continuous exposure. That is, we used 1/5th of the MNU dosage reported in other studies to avoid negatively affecting cell viability in the short term. MNU is an effective nitroso compound for gastric carcinogenesis mouse model construction.^36^, ^37^, ^38^, ^39^, ^40^, ^41^

Nitroso compounds promote the occurrence of malignant tumours by inducing DNA damage and activating downstream pathways that regulate cancer onset and development.^42^, ^43^, ^44^ Innovatively, we explored the role of m6A modification in GC development. Dose‐gradient and time‐course experiments revealed that YTHDF1 is closely related to MNU stimulation of m6A modifiers. *YTHDF1* knockdown suppressed the malignant transformation process induced by MNU. Our findings indicate that long‐term low‐dose MNU exposure induces malignant transformation of cells by enhancing YTHDF1 expression. Meanwhile, inhibition of YTHDF1 expression partially reverses the malignant transformation of cells. m6A modification plays significant roles in physiological, and pathophysiological processes, including carcinogenesis.^21^, ^45^, ^46^, ^47^, ^48^, ^49^, ^50^ As an m6A reader, YTHDF1 is up‐regulated in tumour tissues and related to poor prognosis of various tumours.^24^, ^51^, ^52^, ^53^ YTHDF1 has a regulatory role as a cancer‐promoting factor in the development of GC. According to a previous report, YTHDF1 facilitates GC tumorigenesis and metastasis through mediating USP14 translation in a m6A‐dependent manner.^25^ Another study reported that YTHDF1 mediates Wnt/β‐catenin signalling regulation to promote GC cell proliferation via m6A‐mediated regulation.^24^ Using RNA‐seq and proteomics analyses, we found that differentially expressed genes regulated by YTHDF1 in MNU‐induced transformed cells were enriched in proliferation, stem cell regulation, and cell differentiation. Thus, this study determined, for the first time, that YTHDF1 participates in cell malignant transformation.

Using combined RNA‐seq, proteomics, and RIP, we identified downstream regulation targets of YTHDF1. The m6A sites predicted on SRAMP^33^ and RIP results indicated that YTHDF1 could directly bind to the m6A modification site in *HSPH1* mRNA. In the process of cell transformation, the *HSPH1* transcript level was not significantly altered, whereas its protein abundance was significantly increased and was regulated by YTHDF1. Subsequent experiments demonstrated that HSPH1, as a downstream target of YTHDF1, participated in the cellular transformation process. HSPH1, a member of the mammalian Hsp110 family, promotes the dissociation of protein aggregates to prevent the aggregation of misfolded proteins.^54^, ^55^ More and more evidence suggest that heat stress proteins play crucial roles in tumour progression by interacting with multiple proteins.^55^, ^56^, ^57^, ^58^, ^59^ HSPH1 is overexpressed in various human cancers, including non‐Hodgkin's lymphoma, melanoma, and colon cancer.^60^ It is highly overexpressed in digestive tract cancers which have a high incidence rate in China, and is related to poor prognosis in these patients.^55^ SiRNA‐mediated *HSPH1* knockout induced apoptosis in GC cell lines.^61^ High HSPH1 expression has been found to promote chemoresistance in oral and oesophageal cancers.^55^ This suggests that HSPH1 is a potential drug target for GC. HSPH1 can activate proliferation‐related signalling pathways and transcription factors in tumour cells.^62^ In colon cancer, HSPH1 plays a role in the Wnt/β‐catenin pathway by inhibiting β‐catenin hyperphosphorylation and degradation. This function may contribute to the overall tumorigenic effect of HSPH1. HSPH1 is associated with the occurrence of colon cancer, as it is highly expressed in intestinal epithelial cells and plays a functional role in regulating mucosal homeostasis. Research has shown that HSPH1 plays a functional role in colon cancer cell proliferation through the IL‐6‐STAT3 pathway activation in vitro and in vivo. The function of HSPH1 in regulating protein homeostasis and its role in anti‐apoptosis and promoting proliferation pathways may explain how this protein promotes normal cell malignant transformation. We found that HSPH1 expression was significantly increased in MNU‐stimulated cells, whereas *HSPH1* knockdown partially inhibited the tumorigenic potential of these cells. These findings provide a potential mechanism explanation for nitrosamines in promoting GC occurrence.

In China, GC is closely related to long‐term nitrosamine stimulation. We successfully constructed a long‐term low‐dose nitroso compound‐stimulated gastric mucosal malignant transformation cell model in vitro in 6 months, which can be an effective supplement for the study of nitrosamine‐induced GC in vivo. Using this cell model, we explored the regulatory effect of the m6A‐binding protein YTHDF1 in malignant transformation of gastric epithelial cells. YTHDF1 promotes the proliferation and stem‐cell potential of malignant cells by regulating the expression of the downstream HSPH1 protein. Collectively, this study provided a potential therapeutic target for GC in China.

## AUTHOR CONTRIBUTIONS

**Peng Song:** Conceptualization, Methodology, Writing—original draft, Writing—review & editing. **Xiang Li:** Conceptualization, Methodology, Resources. **Shuai Chen:** Conceptualization, Methodology, Resources. **Yu Gong:** Supervision, Conceptualization. **Jie Zhao:** Data curation, Writing—review & editing. **Yuwen Jiao:** Data curation, Writing—review & editing. **Yi Dai**: Data curation, Writing—review & editing. **Haojun Yang:** Data curation, Writing—review & editing. **Jun Qian:** Resources, Writing—review & editing. **Yuan Li:** Supervision, Writing—review & editing. **Jian He:** Project administration, Writing—review & editing. **Liming Tang:** Project administration, Funding acquisition, Writing—review & editing.

## FUNDING INFORMATION

This work was supported by the Changzhou Sci &Tech Program (CM20223008, CE20215039, 2022CZLJ017, CJ20220155), Changzhou Medical Center of Nanjing Medical University Program (PCMCB202212 & PCMCM202204).

## CONFLICT OF INTEREST STATEMENT

The authors declare that they have no known competing financial interests or personal relationships that could have appeared to influence the work reported in this paper.

## Supporting information


**Data S1:** Supporting information

**Data S2:** Supporting information

## Data Availability

Data will be made available upon request.
