# Correction to “YTHDF1 Mediates N‐Methyl‐N‐Nitrosourea‐Induced Gastric Carcinogenesis by Controlling HSPH1 Translation”

**DOI:** 10.1111/cpr.70076

**Published:** 2025-06-19

P. Song, X. Li, S. Chen, et al., “YTHDF1 Mediates N‐Methyl‐N‐Nitrosourea‐Induced Gastric Carcinogenesis by Controlling HSPH1 Translation,” *Cell Proliferation* 57, no. 7 (2024): e13619, https://doi.org/10.1111/cpr.13619.

1. Error Identification

During post‐publication review, we identified an erroneous image usage in Figure 1D of the Results section. The original figure intended to demonstrate gastric epithelial cell colony formation under N‐methyl‐N‐nitrosourea (MNU) induction was inadvertently replaced with images from other cell experiments in our research.

2 Root Cause Analysis

This error occurred during image organization and labelling. Due to the large volume of experimental images involving multiple cell lines, our team mistakenly cross‐referenced datasets during figure compilation. The oversight persisted through internal quality checks prior to submission.

3 Impact Assessment

While the core conclusions of our study remain fully supported by validated data, we acknowledge that this misrepresentation compromises methodological transparency. We deeply regret any confusion this may have caused readers and the scientific community. Our reanalysed results from the revised images demonstrate that MNU‐induced colony formation in GES‐1 cells showed no significant difference compared to the control group at the 3‐month time point, whereas statistically significant differences were observed at the 6‐month time point. While this temporal distinction does not impact the core conclusions of our study, we believe it is imperative to present the authentic experimental findings to our readers.

Corrected Figure 1 is provided below.

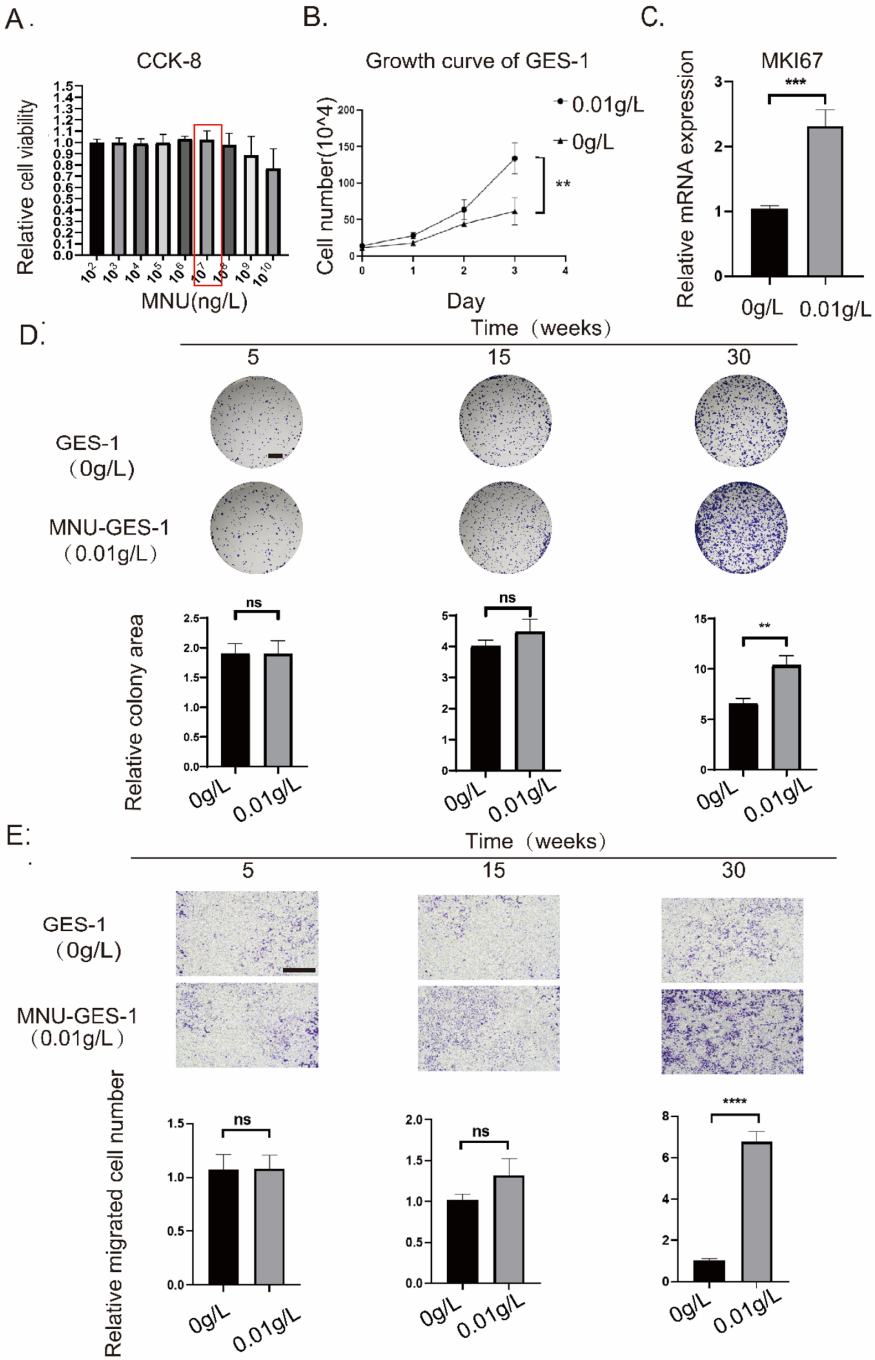

We apologise for this error.

