# Supplementary material for: YTHDF1 mediates N‐methyl‐N ‐nitrosourea‐induced gastric carcinogenesis by controlling HSPH1 translation
Source: Cell Prolif. 2024 Mar 5;57(7):e13619. doi: 10.1111/cpr.13619 (PMC11216948; doi:10.1111/cpr.13619)
Supplement: Supplementary file 2 — Data S2: Supporting information [file CPR-57-e13619-s001.docx]

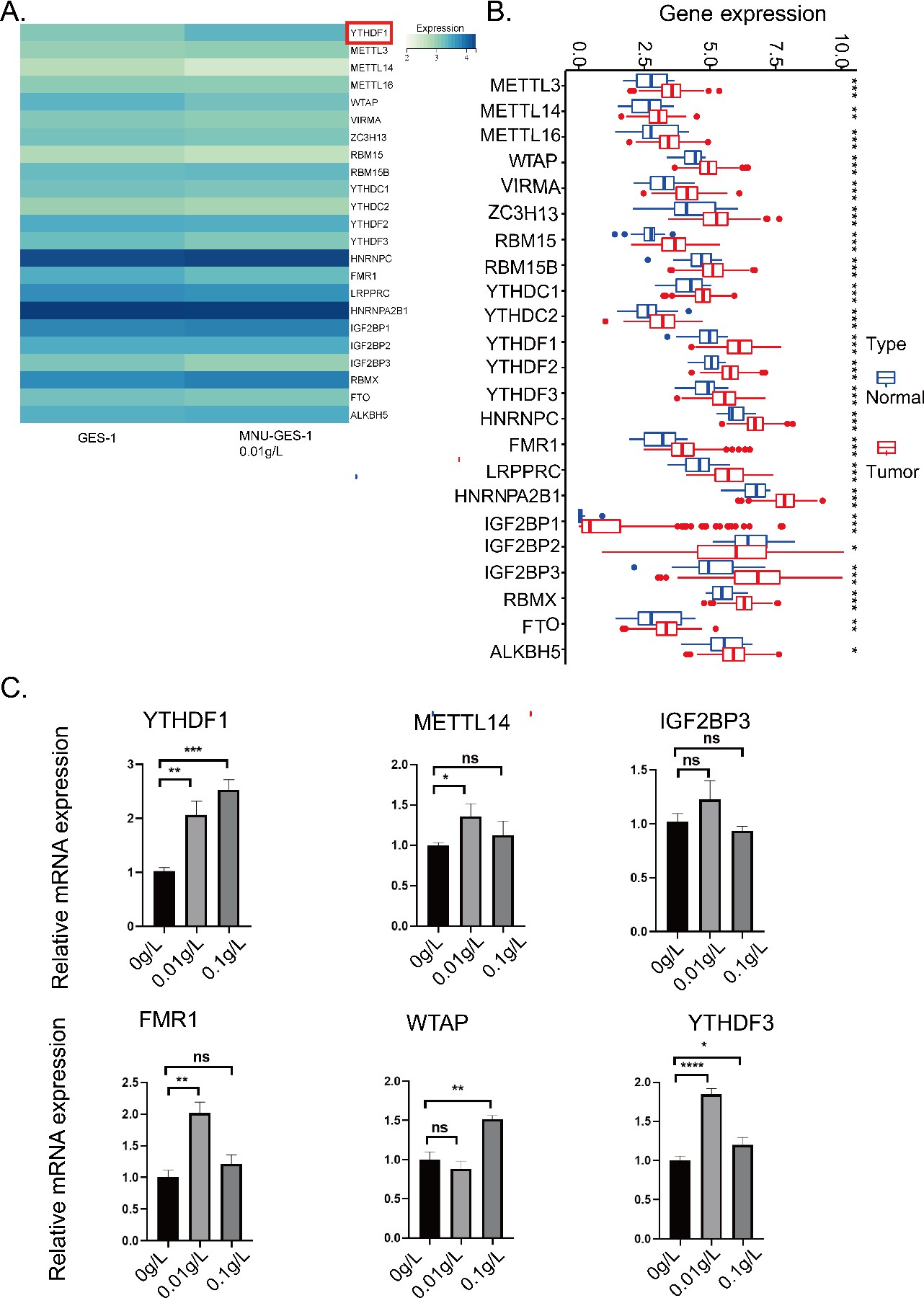


Figure S1: Relationship between YTHDF1 expression and MNU stimulation. A: Relative expression changes in 23 m6A regulators in GC tissues compared to matched normal tissues according to TCGA datasets. B: Western blot analysis of YTHDF1 expression in MNU-induced cells at different time points. C: qPCR and western blot

analyses of YTHDF1 expression in GES-1 cells treated with different concentrations of MNU for 48 h. D: qPCR and western blot analyses of YTHDF1 expression in GES-1 cells treated with 0.1 g/L MNU for different periods. ns P > 0.05, *P < 0.05,

**P < 0.01, ***P < 0.001, ****P < 0.0001


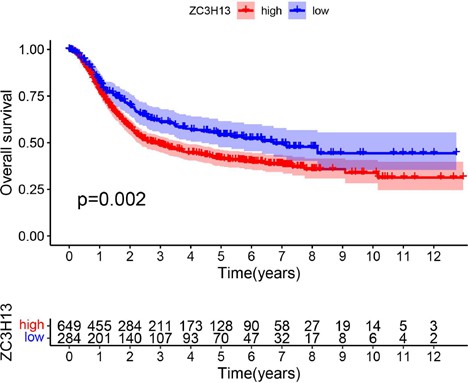

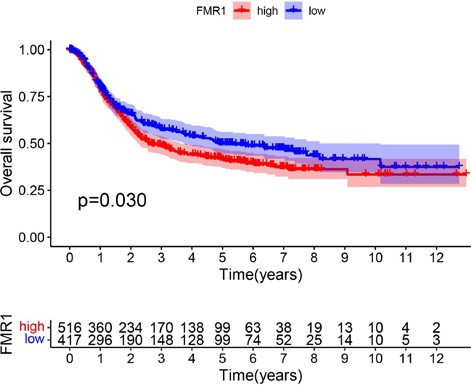

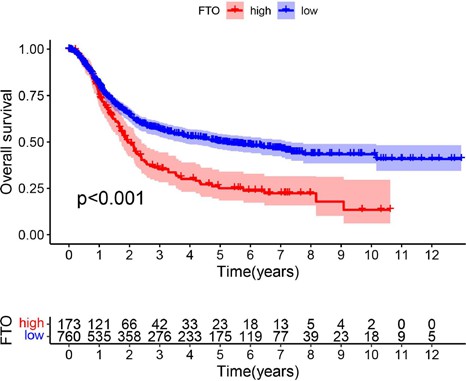

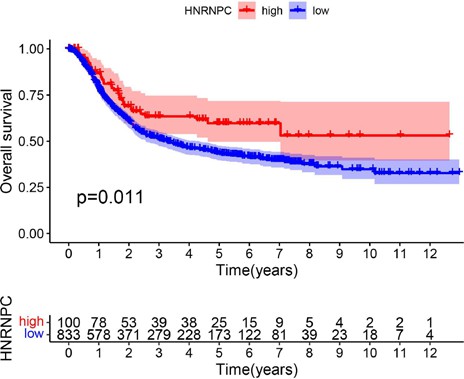


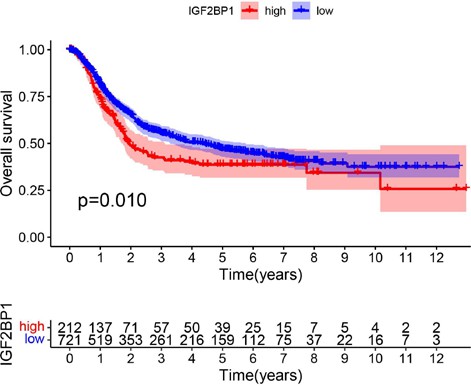

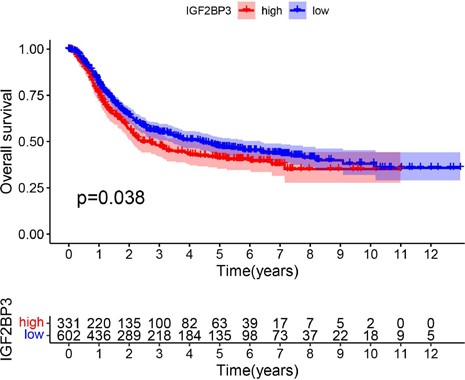

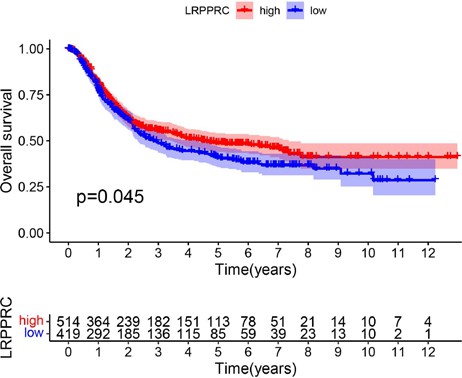


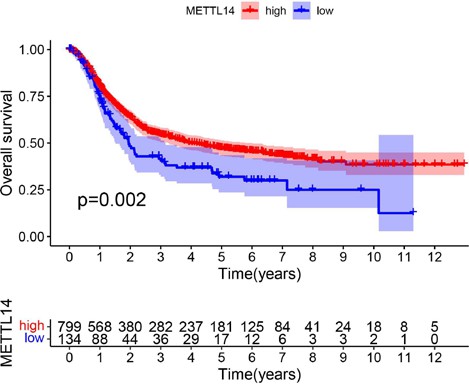

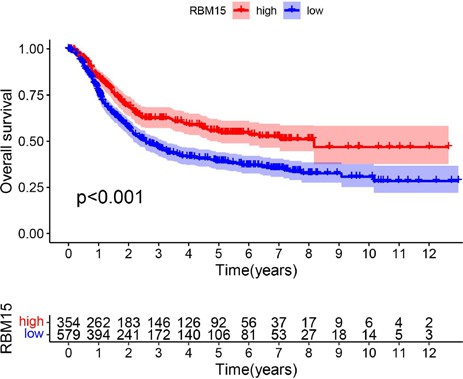

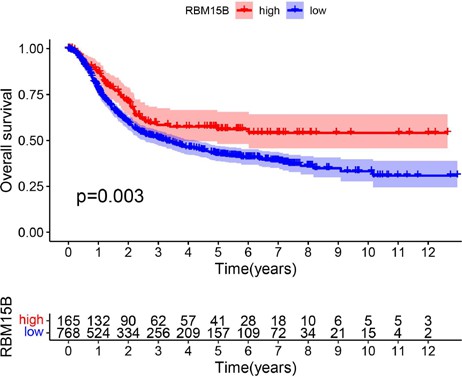


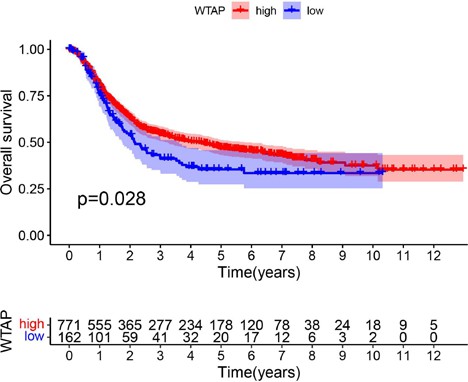

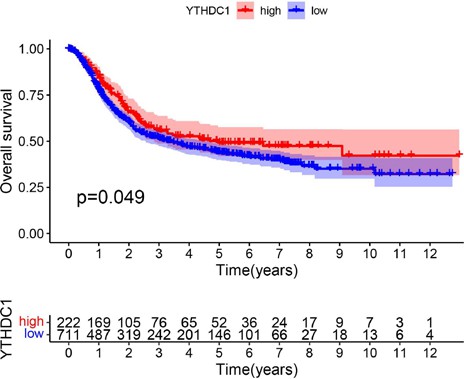

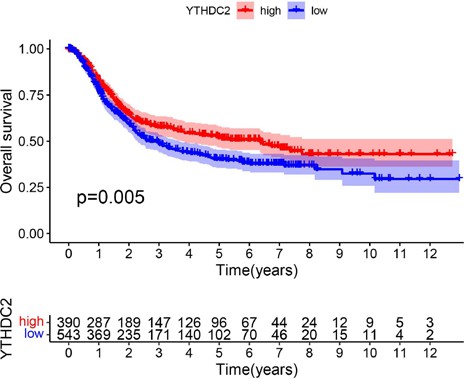

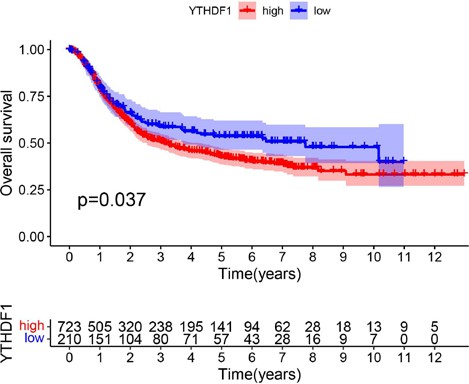

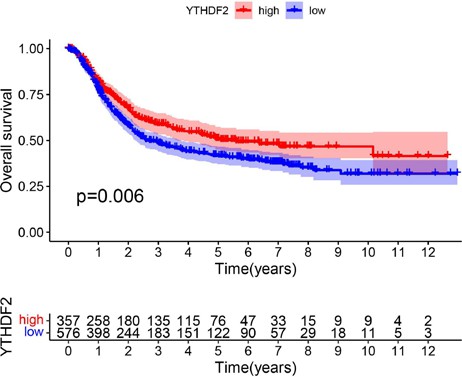

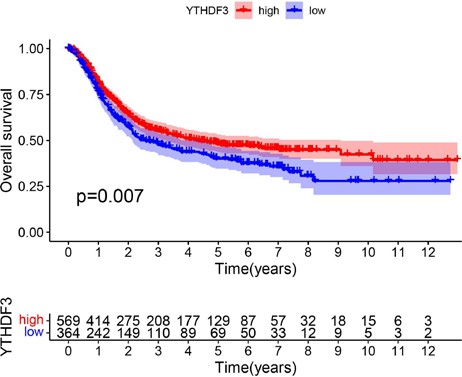


16

Figure S2: Correlation between the expression of m6A regulator genes and prognosis. 21 of 23 m6A regulators are relevant to the prognosis of tumors.


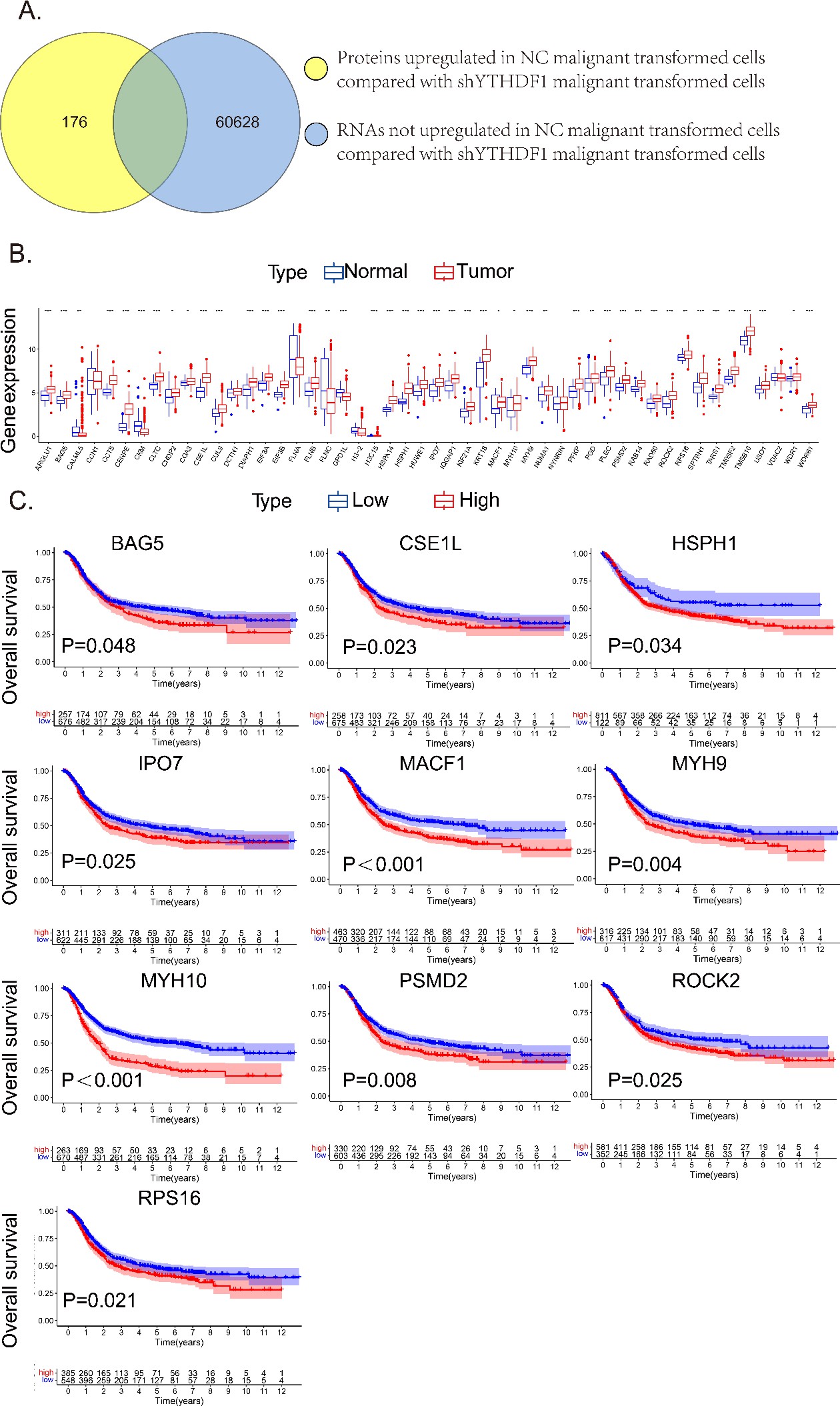


Figure S3: Analysis of potential downstream target genes of YTHDF1 reveals that HSPH1 is a m6A-modification target of YTHDF1. A: Potential YTHDF1 targets authenticated by combined RNA-seq and proteomics analysis of control GES-1 cells and shYTHDF1 MNU-induced transformed cells. B: Expression of candidate target genes in GC patients based on TCGA dataset. C: Kaplan–Meier curves for ten candidate targets based on the four GEO and TCGA datasets.


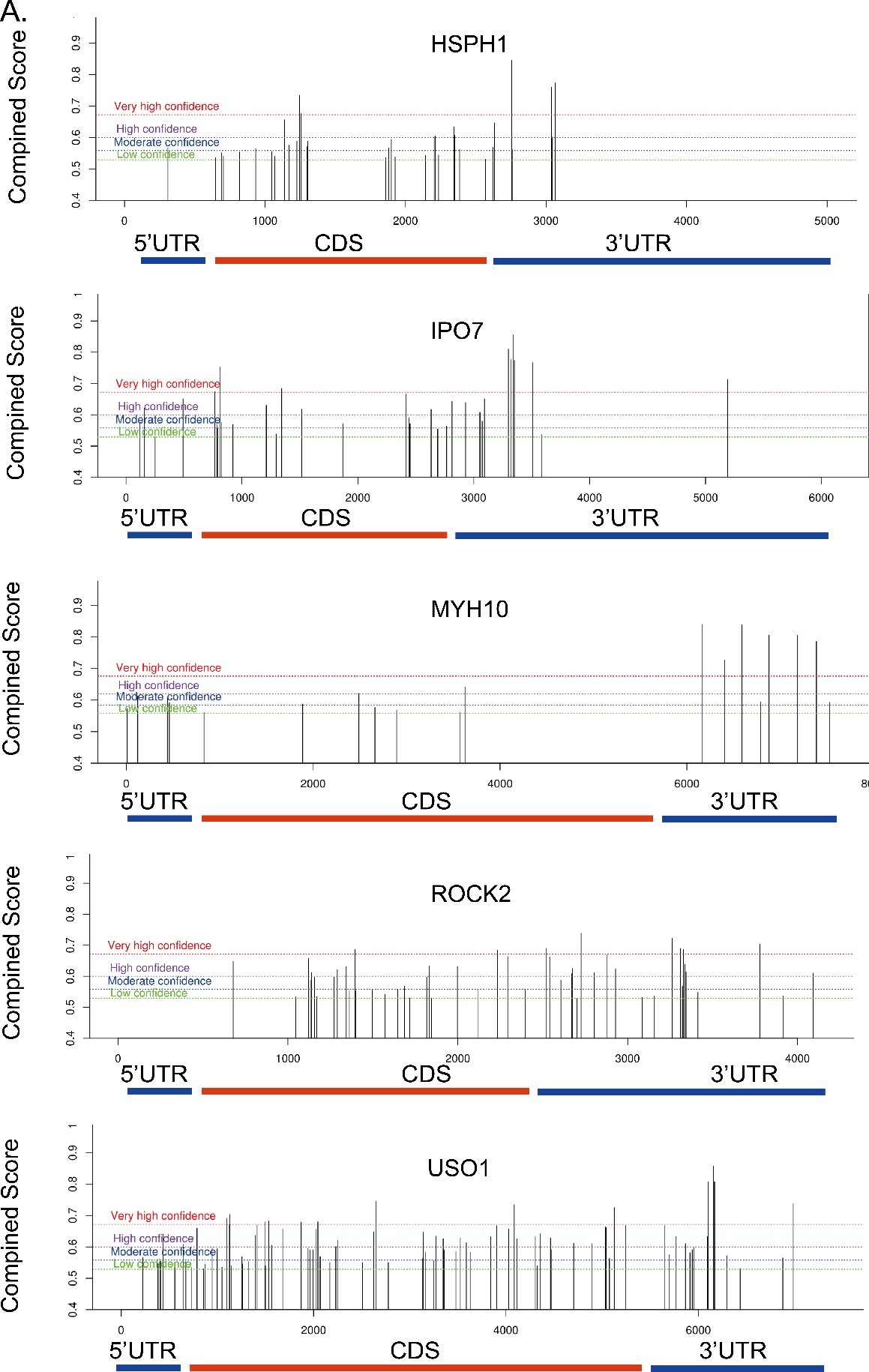


B.


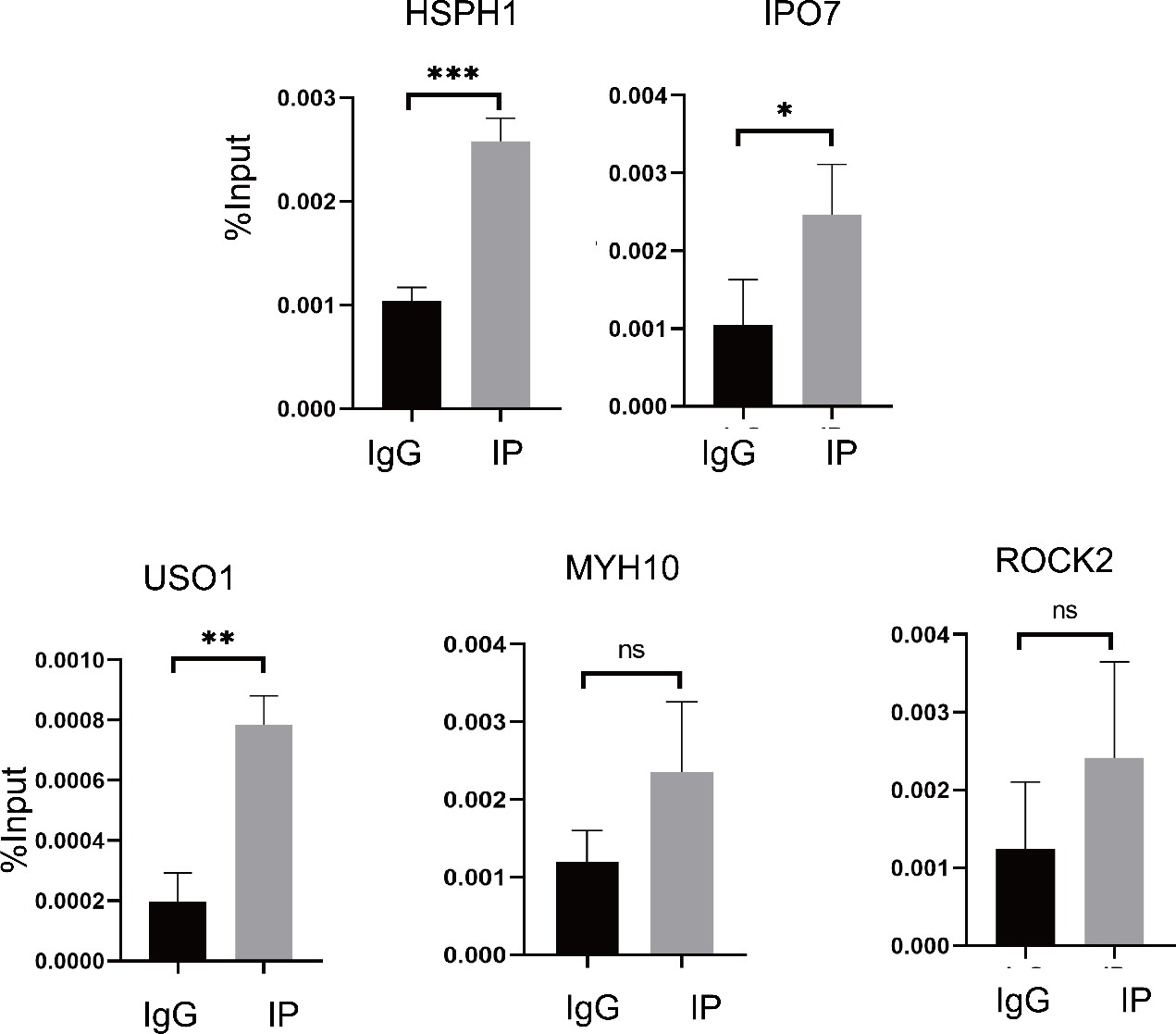


Figure S4: HSPH1 is a m6A modification target of YTHDF1. A: The SRAMP program was used to predict m6A sites in transcripts, particularly in the 3′-UTR. B: YTHDF1-RIP-qPCR confirmed the interaction between YTHDF1 and transcripts in MNU-induced transformed cells.


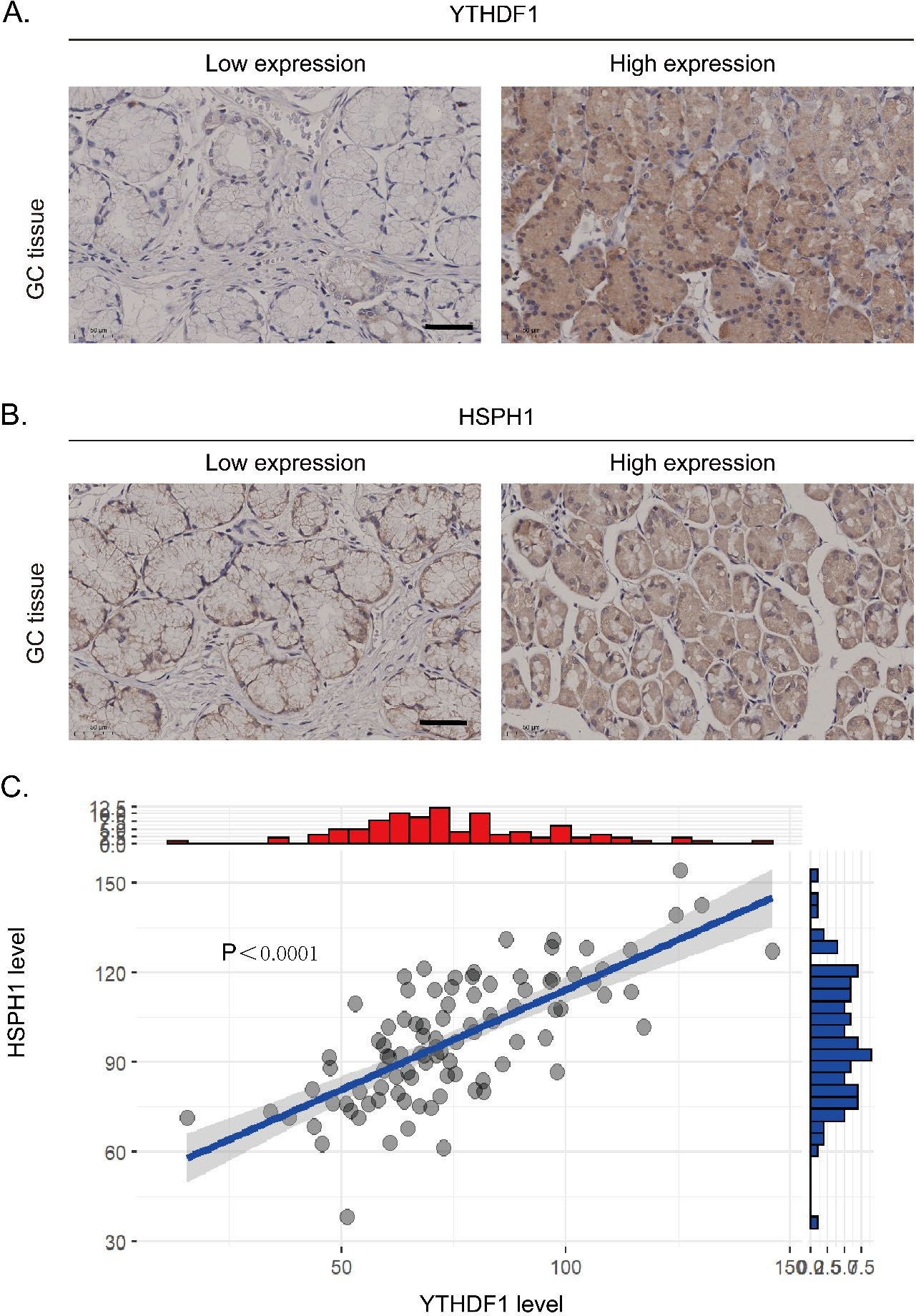


Figure S5: Correlation analysis of protein levels between YTHDF1 and HSPH1 in 48 cases of clinical samples. A：Immunohistochemical staining for YTHDF1 in gastric

cancer tissue microarray. indicate weakly positive staining. Left: Weakly positive staining for YTHDF1.Right: Strongly positive staining for YTHDF1.B ： Immunohistochemical staining for HSPH1 in gastric cancer tissue microarray. indicate weakly positive staining. Left: Weakly positive staining for HSPH1.Right: Strongly positive staining for HSPH1. Black line = 50μm. C：Linear correlation statistics between YTHDF1 and HSPH1 immunohistochemistry score (Histochemistry score).The abscissa represents the YTHDF1 score, while the ordinate represents the HSPH1 score.
